# A Meta-Analysis of the Impacts of Genetically Modified Crops

**DOI:** 10.1371/journal.pone.0111629

**Published:** 2014-11-03

**Authors:** Wilhelm Klümper, Matin Qaim

**Affiliations:** Department of Agricultural Economics and Rural Development, Georg-August-University of Goettingen, Goettingen, Germany; University of Perugia, Italy

## Abstract

**Background:**

Despite the rapid adoption of genetically modified (GM) crops by farmers in many countries, controversies about this technology continue. Uncertainty about GM crop impacts is one reason for widespread public suspicion.

**Objective:**

We carry out a meta-analysis of the agronomic and economic impacts of GM crops to consolidate the evidence.

**Data Sources:**

Original studies for inclusion were identified through keyword searches in ISI Web of Knowledge, Google Scholar, EconLit, and AgEcon Search.

**Study Eligibility Criteria:**

Studies were included when they build on primary data from farm surveys or field trials anywhere in the world, and when they report impacts of GM soybean, maize, or cotton on crop yields, pesticide use, and/or farmer profits. In total, 147 original studies were included.

**Synthesis Methods:**

Analysis of mean impacts and meta-regressions to examine factors that influence outcomes.

**Results:**

On average, GM technology adoption has reduced chemical pesticide use by 37%, increased crop yields by 22%, and increased farmer profits by 68%. Yield gains and pesticide reductions are larger for insect-resistant crops than for herbicide-tolerant crops. Yield and profit gains are higher in developing countries than in developed countries.

**Limitations:**

Several of the original studies did not report sample sizes and measures of variance.

**Conclusion:**

The meta-analysis reveals robust evidence of GM crop benefits for farmers in developed and developing countries. Such evidence may help to gradually increase public trust in this technology.

## Introduction

Despite the rapid adoption of genetically modified (GM) crops by farmers in many countries, public controversies about the risks and benefits continue [1]–[4]. Numerous independent science academies and regulatory bodies have reviewed the evidence about risks, concluding that commercialized GM crops are safe for human consumption and the environment [5]–[7]. There are also plenty of studies showing that GM crops cause benefits in terms of higher yields and cost savings in agricultural production [8]–[12], and welfare gains among adopting farm households [13]–[15]. However, some argue that the evidence about impacts is mixed and that studies showing large benefits may have problems with the data and methods used [16]–[18]. Uncertainty about GM crop impacts is one reason for the widespread public suspicion towards this technology. We have carried out a meta-analysis that may help to consolidate the evidence.

While earlier reviews of GM crop impacts exist [19]–[22], our approach adds to the knowledge in two important ways. First, we include more recent studies into the meta-analysis. In the emerging literature on GM crop impacts, new studies are published continuously, broadening the geographical area covered, the methods used, and the type of outcome variables considered. For instance, in addition to other impacts we analyze effects of GM crop adoption on pesticide quantity, which previous meta-analyses could not because of the limited number of observations for this particular outcome variable. Second, we go beyond average impacts and use meta-regressions to explain impact heterogeneity and test for possible biases.

Our meta-analysis concentrates on the most important GM crops, including herbicide-tolerant (HT) soybean, maize, and cotton, as well as insect-resistant (IR) maize and cotton. For these crops, a sufficiently large number of original impact studies have been published to estimate meaningful average effect sizes. We estimate mean impacts of GM crop adoption on crop yield, pesticide quantity, pesticide cost, total production cost, and farmer profit. Furthermore, we analyze several factors that may influence outcomes, such as geographic location, modified crop trait, and type of data and methods used in the original studies.

## Materials and Methods

### Literature search

Original studies for inclusion in this meta-analysis were identified through keyword searches in relevant literature databanks. Studies were searched in the ISI Web of Knowledge, Google Scholar, EconLit, and AgEcon Search. We searched for studies in the English language that were published after 1995. We did not extend the review to earlier years, because the commercial adoption of GM crops started only in the mid-1990s [23]. The search was performed for combinations of keywords related to GM technology and related to the outcome of interest. Concrete keywords used related to GM technology were (an asterisk is a replacement for any ending of the respective term; quotation marks indicate that the term was used as a whole, not each word alone): GM*, “genetically engineered”, “genetically modified”, transgenic, “agricultural biotechnology”, HT, “herbicide tolerant”, Roundup, Bt, “insect resistant”. Concrete keywords used related to outcome variables were: impact*, effect*, benefit*, yield*, economic*, income*, cost*, soci*, pesticide*, herbicide*, insecticide*, productivity*, margin*, profit*. The search was completed in March 2014.

Most of the publications in the ISI Web of Knowledge are articles in academic journals, while Google Scholar, EconLit, and AgEcon Search also comprise book chapters and grey literature such as conference papers, working papers, and reports in institutional series. Articles published in academic journals have usually passed a rigorous peer-review process. Most papers presented at academic conferences have also passed a peer-review process, which is often less strict than that of good journals though. Some of the other publications are peer reviewed, while many are not. Some of the working papers and reports are published by research institutes or government organizations, while others are NGO publications. Unlike previous reviews of GM crop impacts, we did not limit the sample to peer-reviewed studies but included all publications for two reasons. First, a clear-cut distinction between studies with and without peer review is not always possible, especially when dealing with papers that were not published in a journal or presented at an academic conference [24]. Second, studies without peer review also influence the public and policy debate on GM crops; ignoring them completely would be short-sighted.

Of the studies identified through the keyword searches, not all reported original impact results. We classified studies by screening titles, abstracts, and full texts. Studies had to fulfill the following criteria to be included:

- The study is an empirical investigation of the agronomic and/or economic impacts of GM soybean, GM maize, or GM cotton using micro-level data from individual plots and/or farms. Other GM crops such as GM rapeseed, GM sugarbeet, and GM papaya were commercialized in selected countries [23], but the number of impact studies available for these other crops is very small.
- The study reports GM crop impacts in terms of one or more of the following outcome variables: yield, pesticide quantity (especially insecticides and herbicides), pesticide costs, total variable costs, gross margins, farmer profits. If only the number of pesticide sprays was reported, this was used as a proxy for pesticide quantity.
- The study analyzes the performance of GM crops by either reporting mean outcomes for GM and non-GM, absolute or percentage differences, or estimated coefficients of regression models that can be used to calculate percentage differences between GM and non-GM crops.
- The study contains original results and is not only a review of previous studies.

**Figure 1:**
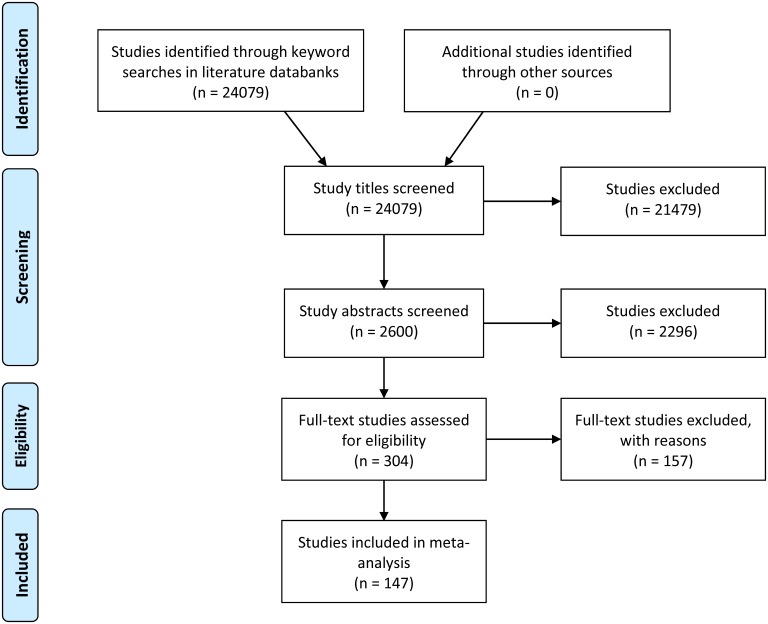
Selection of studies for inclusion in the meta-analysis.

In some cases, the same results were reported in different publications; in these cases, only one of the publications was included to avoid double counting. On the other hand, several publications involve more than one impact observation, even for a single outcome variable, for instance when reporting results for different geographical regions or derived with different methods (e.g., comparison of mean outcomes of GM and non-GM crops plus regression model estimates). In those cases, all observations were included. Moreover, the same primary dataset was sometimes used for different publications without reporting identical results (e.g., analysis of different outcome variables, different waves of panel data, use of different methods). Hence, the number of impact observations in our sample is larger than the number of publications and primary datasets (Data S1). The number of studies selected at various stages is shown in the flow diagram in Figure 1. The number of publications finally included in the meta-analysis is 147 (Table S1).

### Effect sizes and influencing factors

Effect sizes are measures of outcome variables. We chose the percentage difference between GM and non-GM crops for five different outcome variables, namely yield, pesticide quantity, pesticide cost, total production cost, and farmer profits per unit area. Most studies that analyze production costs focus on variable costs, which are the costs primarily affected through GM technology adoption. Accordingly, profits are calculated as revenues minus variable production costs (profits calculated in this way are also referred to as gross margins). These production costs also take into account the higher prices charged by private companies for GM seeds. Hence, the percentage differences in profits considered here are net economic benefits for farmers using GM technology. Percentage differences, when not reported in the original studies, were calculated from mean value comparisons between GM and non-GM or from estimated regression coefficients.

Since we look at different types of GM technologies (different modified traits) that are used in different countries and regions, we do not expect that effect sizes are homogenous across studies. Hence, our approach of combining effect sizes corresponds to a random-effects model in meta-analysis [25]. To explain impact heterogeneity and test for possible biases, we also compiled data on a number of study descriptors that may influence the reported effect sizes. These influencing factors include information on the type of GM technology (modified trait), the region studied, the type of data and method used, the source of funding, and the type of publication. All influencing factors are defined as dummy variables. The exact definition of these dummy variables is given in Table 1. Variable distributions of the study descriptors are shown in Table S2.

**Table 1:** Variables used to analyze influencing factors of GM crop impacts.

Variable name	Variable definition
Insect resistance(IR)	Dummy that takes a value of one for all observations referring to insect-resistant GM crops with genes from *Bacillus thuringiensis* (Bt), and zero for all herbicide-tolerant (HT) GM crops.
Developing country	Dummy that takes a value of one for all GM crop applications in a developing country according to the World Bank classification of countries, and zero for all applications in a developed country.
Field-trial data	Dummy that takes a value of one for all observations building on field-trial data (on-station and on-farm experiments), and zero for all observations building on farm survey data.
Industry-fundedstudy	Dummy that takes a value of one for all studies that mention industry (private sector companies) as source of funding, and zero otherwise.
Regression modelresult	Dummy that takes a value of one for all impact observations that are derived from regression model estimates, and zero for observations derived from mean value comparisons between GM and non-GM.
Journal publication	Dummy that takes a value of one for all studies published in a peer-reviewed journal, and zero otherwise.
Journal/academicconference	Dummy that takes a value of one for all studies published in a peer-reviewed journal or presented at an academic conference, and zero otherwise.

### Statistical analysis

In a first step, we estimate average effect sizes for each outcome variable. To test whether these mean impacts are significantly different from zero, we regress each outcome variable on a constant with cluster correction of standard errors by primary dataset. Thus, the test for significance is valid also when observations from the same dataset are correlated. We estimate average effect sizes for all GM crops combined. However, we expect that the results may differ by modified trait, so that we also analyze mean effects for HT crops and IR crops separately.

Meta-analyses often weight impact estimates by their variances; estimates with low variance are considered more reliable and receive a higher weight [26]. In our case, several of the original studies do not report measures of variance, so that weighting by variance is not possible. Alternatively, weighting by sample size is common, but sample sizes are also not reported in all studies considered, especially not in some of the grey literature publications. To test the robustness of the results, we employ a different weighting procedure, using the inverse of the number of impact observations per dataset as weights. This procedure avoids that individual datasets that were used in several publications dominate the calculation of average effect sizes.

In a second step, we use meta-regressions to explain impact heterogeneity and test for possible biases. Linear regression models are estimated separately for all of the five outcome variables:
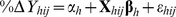


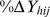
 is the effect size (percentage difference between GM and non-GM) of each outcome variable *h* for observation *i* in publication *j*, and 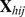 is a vector of influencing factors. 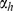 is a coefficient and 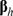 a vector of coefficients to be estimated; 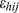 is a random error term. Influencing factors used in the regressions are defined in Table 1.

## Results and Discussion

### Average effect sizes

Distributions of all five outcome variables are shown in Figure S1. Table 2 presents unweighted mean impacts. As a robustness check, we weighted by the inverse of the number of impact observations per dataset. Comparing unweighted results (Table 2) with weighted results (Table S3) we find only very small differences. This comparison suggests that the unweighted results are robust.

**Table 2:** Impacts of GM crop adoption by modified trait.

Outcome variable	All GM crops	Insect resistance	Herbicide tolerance
Yield	21.57***(15.65; 27.48)	24.85***(18.49; 31.22)	9.29**(1.78; 16.80)
* n*/*m*	451/100	353/83	94/25
Pesticide quantity	–36.93***(–48.01; −25.86)	–41.67***(–51.99; −31.36)	2.43(–20.26; 25.12)
* n*/*m*	121/37	108/31	13/7
Pesticide cost	–39.15***(–46.96; −31.33)	–43.43***(–51.64; −35.22)	–25.29***(–33.84; −16.74)
* n*/*m*	193/57	145/45	48/15
Total productioncost	3.25(–1.76; 8.25)	5.24**(0.25; 10.73)	–6.83(–16.43; 2.77)
* n*/*m*	115/46	96/38	19/10
Farmer profit	68.21***(46.31; 90.12)	68.78***(46.45; 91.11)	64.29(–24.73; 153.31)
* n*/*m*	136/42	119/36	17/9

Average percentage differences between GM and non-GM crops are shown with 95% confidence intervals in parentheses. *, **, *** indicate statistical significance at the 10%, 5%, and 1% level, respectively. *n* is the number of observations, *m* the number of different primary datasets from which these observations are derived.

**Figure 2:**
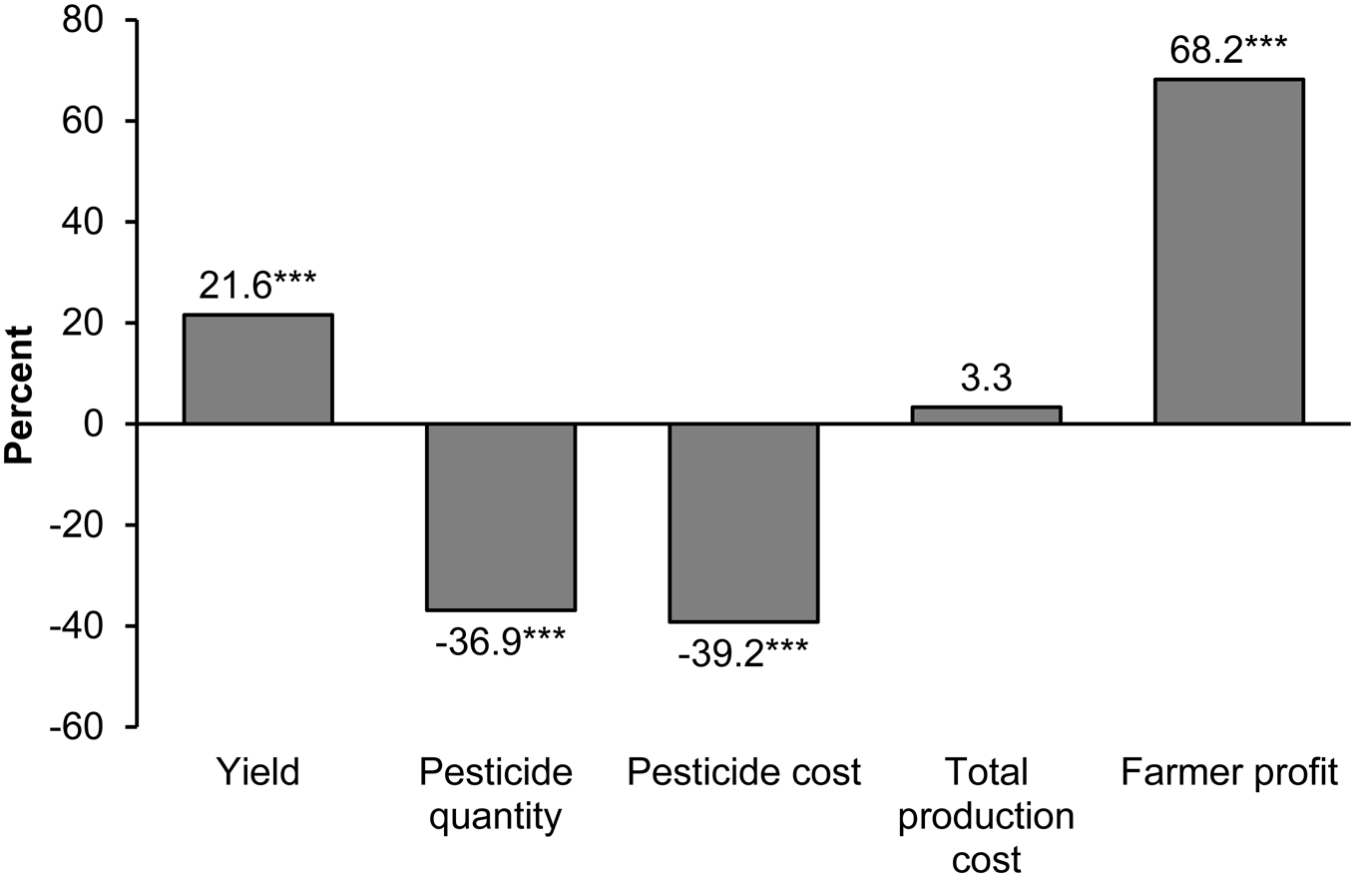
Impacts of GM crop adoption. Average percentage differences between GM and non-GM crops are shown. Results refer to all GM crops, including herbicide-tolerant and insect-resistant traits. The number of observations varies by outcome variable; yield: 451; pesticide quantity: 121; pesticide cost: 193; total production cost: 115; farmer profit: 136. *** indicates statistical significance at the 1% level.

On average, GM technology has increased crop yields by 21% (Figure 2). These yield increases are not due to higher genetic yield potential, but to more effective pest control and thus lower crop damage [27]. At the same time, GM crops have reduced pesticide quantity by 37% and pesticide cost by 39%. The effect on the cost of production is not significant. GM seeds are more expensive than non-GM seeds, but the additional seed costs are compensated through savings in chemical and mechanical pest control. Average profit gains for GM-adopting farmers are 69%.

Results of Cochran’s test [25], which are reported in Figure S1, confirm that there is significant heterogeneity across study observations for all five outcome variables. Hence it is useful to further disaggregate the results. Table 2 shows a breakdown by modified crop trait. While significant reductions in pesticide costs are observed for both HT and IR crops, only IR crops cause a consistent reduction in pesticide quantity. Such disparities are expected, because the two technologies are quite different. IR crops protect themselves against certain insect pests, so that spraying can be reduced. HT crops, on the other hand, are not protected against pests but against a broad-spectrum chemical herbicide (mostly glyphosate), use of which facilitates weed control. While HT crops have reduced herbicide quantity in some situations, they have contributed to increases in the use of broad-spectrum herbicides elsewhere [2], [11], [19]. The savings in pesticide costs for HT crops in spite of higher quantities can be explained by the fact that broad-spectrum herbicides are often much cheaper than the selective herbicides that were used before. The average farmer profit effect for HT crops is large and positive, but not statistically significant because of considerable variation and a relatively small number of observations for this outcome variable.

### Impact heterogeneity and possible biases

Table 3 shows the estimation results from the meta-regressions that explain how different factors influence impact heterogeneity. Controlling for other factors, yield gains of IR crops are almost 7 percentage points higher than those of HT crops (column 1). Furthermore, yield gains of GM crops are 14 percentage points higher in developing countries than in developed countries. Especially smallholder farmers in the tropics and subtropics suffer from considerable pest damage that can be reduced through GM crop adoption [27].

**Table 3:** Factors influencing results on GM crop impacts (%).

	(1)	(2)	(3)	(4)	(5)	(6)	(7)
Variables	Yield	Yield	Pesticidequantity	Pesticidecost	Totalcost	Farmerprofit	Farmerprofit
Insect resistance(IR)	6.58**(2.85)	5.25*(2.82)	–37.38***(11.81)	–7.28(5.44)	5.63(5.60)	–22.33(21.62)	–33.41(21.94)
Developing country	14.17***(2.72)	13.32***(2.65)	–10.23(8.99)	–19.16***(5.35)	3.43(4.78)	59.52***(18.02)	60.58***(17.67)
Field-trial data	–7.14**(3.19)	–7.81**(3.08)	–^#^	–17.56(11.45)	–10.69*(5.79)	–^#^	–^#^
Industry-fundedstudy	1.68(5.30)	1.05(5.21)	37.04(23.08)	–7.77(10.22)	–^#^	–^#^	–^#^
Regression modelresult	7.38*(3.90)	7.29*(3.83)	9.67(10.40)	–^#^	–^#^	–11.44(24.33)	–9.85(24.03)
Journal publication	12.00***(2.52)	–	9.95(6.79)	–3.71(4.09)	–3.08(3.30)	48.27***(15.48)	–
Journal/academicconference	–	16.48***(2.64)	–	–	–	–	65.29***(17.75)
Constant	–0.22(2.84)	–2.64(2.86)	–4.44(10.33)	–16.13(4.88)	–1.02(4.86)	8.57(24.33)	–1.19(24.53)
Observations	451	451	121	193	115	136	136
R^2^	0.23	0.25	0.20	0.14	0.12	0.12	0.14

Coefficient estimates from linear regression models are shown with standard errors in parentheses. Dependent variables are GM crop impacts measured as percentage differences between GM and non-GM. All explanatory variables are 0/1 dummies (for variable definitions see Table 1). The yield models in columns (1) and (2) and the farmer profit models in columns (6) and (7) have the same dependent variables, but they differ in terms of the explanatory variables, as shown. *, **, *** indicate statistical significance at the 10%, 5%, and 1% level, respectively. ^#^ indicates that the variable was dropped because the number of observations with a value of one was smaller than 5.

Most original studies in this meta-analysis build on farm surveys, although some are based on field-trial data. Field-trial results are often criticized to overestimate impacts, because farmers may not be able to replicate experimental conditions. However, results in Table 3 (column 1) show that field-trial data do not overestimate the yield effects of GM crops. Reported yield gains from field trials are even lower than those from farm surveys. This is plausible, because pest damage in non-GM crops is often more severe in farmers’ fields than on well-managed experimental plots.

Another concern often voiced in the public debate is that studies funded by industry money might report inflated benefits. Our results show that the source of funding does not significantly influence the impact estimates. We also analyzed whether the statistical method plays a role. Many of the earlier studies just compared yields of GM and non-GM crops without considering possible differences in other inputs and conditions that may also affect the outcome. Net impacts of GM technology can be estimated with regression-based production function models that control for other factors. Interestingly, results derived from regression analysis report higher average yield effects.

Finally, we examined whether the type of publication matters. Controlling for other factors, the regression coefficient for journal publications in column (1) of Table 3 implies that studies published in peer-reviewed journals show 12 percentage points higher yield gains than studies published elsewhere. Indeed, when only including observations from studies that were published in journals, the mean effect size is larger than if all observations are included (Figure S2). On first sight, one might suspect publication bias, meaning that only studies that report substantial effects are accepted for publication in a journal. A common way to assess possible publication bias in meta-analysis is through funnel plots [25], which we show in Figure S3. However, in our case these funnel plots should not be over-interpreted. First, only studies that report variance measures can be included in the funnel plots, which holds true only for a subset of the original studies used here. Second, even if there were publication bias, our mean results would be estimated correctly, because we do include studies that were not published in peer-reviewed journals.

Further analysis suggests that the journal review process does not systematically filter out studies with small effect sizes. The journal articles in the sample report a wide range of yield effects, even including negative estimates in some cases. Moreover, when combining journal articles with papers presented at academic conferences, average yield gains are even higher (Table 3, column 2). Studies that were neither published in a journal nor presented at an academic conference encompass a diverse set of papers, including reports by NGOs and outspoken biotechnology critics. These reports show lower GM yield effects on average, but not all meet common scientific standards. Hence, rather than indicating publication bias, the positive and significant journal coefficient may be the result of a negative NGO bias in some of the grey literature.

Concerning other outcome variables, IR crops have much stronger reducing effects on pesticide quantity than HT crops (Table 3, column 3), as already discussed above. In terms of pesticide costs, the difference between IR and HT is less pronounced and not statistically significant (column 4). The profit gains of GM crops are 60 percentage points higher in developing countries than in developed countries (column 6). This large difference is due to higher GM yield gains and stronger pesticide cost savings in developing countries. Moreover, most GM crops are not patented in developing countries, so that GM seed prices are lower [19]. Like for yields, studies published in peer-reviewed journals report higher profit gains than studies published elsewhere, but again we do not find evidence of publication bias (column 7).

## Conclusion

This meta-analysis confirms that – in spite of impact heterogeneity – the average agronomic and economic benefits of GM crops are large and significant. Impacts vary especially by modified crop trait and geographic region. Yield gains and pesticide reductions are larger for IR crops than for HT crops. Yield and farmer profit gains are higher in developing countries than in developed countries. Recent impact studies used better data and methods than earlier studies, but these improvements in study design did not reduce the estimates of GM crop advantages. Rather, NGO reports and other publications without scientific peer review seem to bias the impact estimates downward. But even with such biased estimates included, mean effects remain sizeable.

One limitation is that not all of the original studies included in this meta-analysis reported sample sizes and measures of variance. This is not untypical for analyses in the social sciences, especially when studies from the grey literature are also included. Future impact studies with primary data should follow more standardized reporting procedures. Nevertheless, our findings reveal that there is robust evidence of GM crop benefits. Such evidence may help to gradually increase public trust in this promising technology.

## Supporting Information

Figure S1 **Histograms of effect sizes for the five outcome variables.**
(PDF)

Figure S2 **Impacts of GM crop adoption including only studies published in journals.**
(PDF)

Figure S3 **Funnel plots for the five outcome variables.**
(PDF)

Table S1 **List of publications included in the meta-analysis.**
(PDF)

Table S2 **Distribution of study descriptor dummy variables for different outcomes.**
(PDF)

Table S3 **Weighted mean impacts of GM crop adoption.**
(PDF)

Data S1 **Data used for the meta-analysis.**
(PDF)
