# Supplementary material for: A Meta-Analysis of the Impacts of Genetically Modified Crops
Source: PLoS One. 2014 Nov 3;9(11):e111629. doi: 10.1371/journal.pone.0111629 (PMC4218791; doi:10.1371/journal.pone.0111629)
Supplement: Figure S1 — Histograms of effect sizes for the five outcome variables. (PDF) [file pone.0111629.s001.pdf]

Figure S1. Histograms of effect sizes for the five outcome variables

a) Change in yield through GM crop adoption (451 observations)

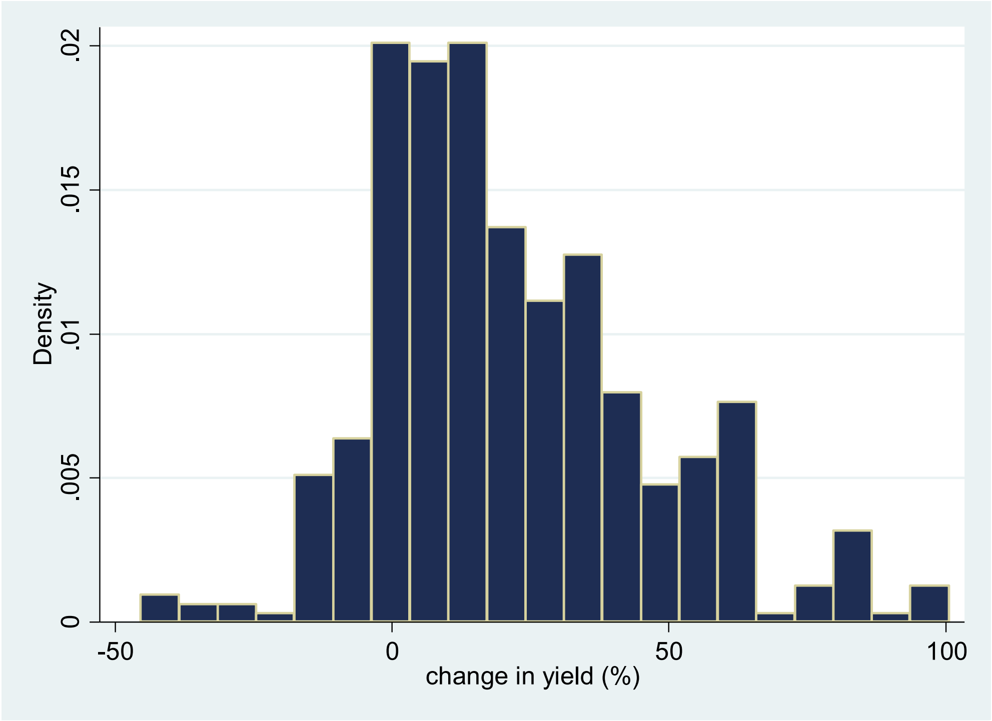

b) Change in pesticide quantity through GM crop adoption (121 observations)

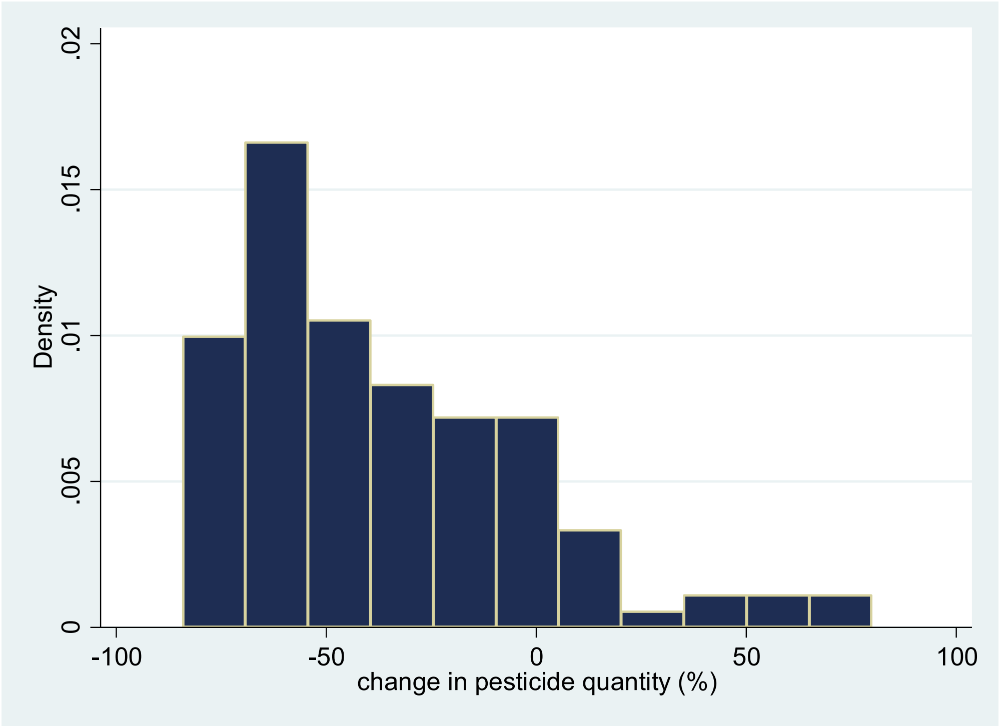

c) Change in pesticide cost through GM crop adoption (193 observations)

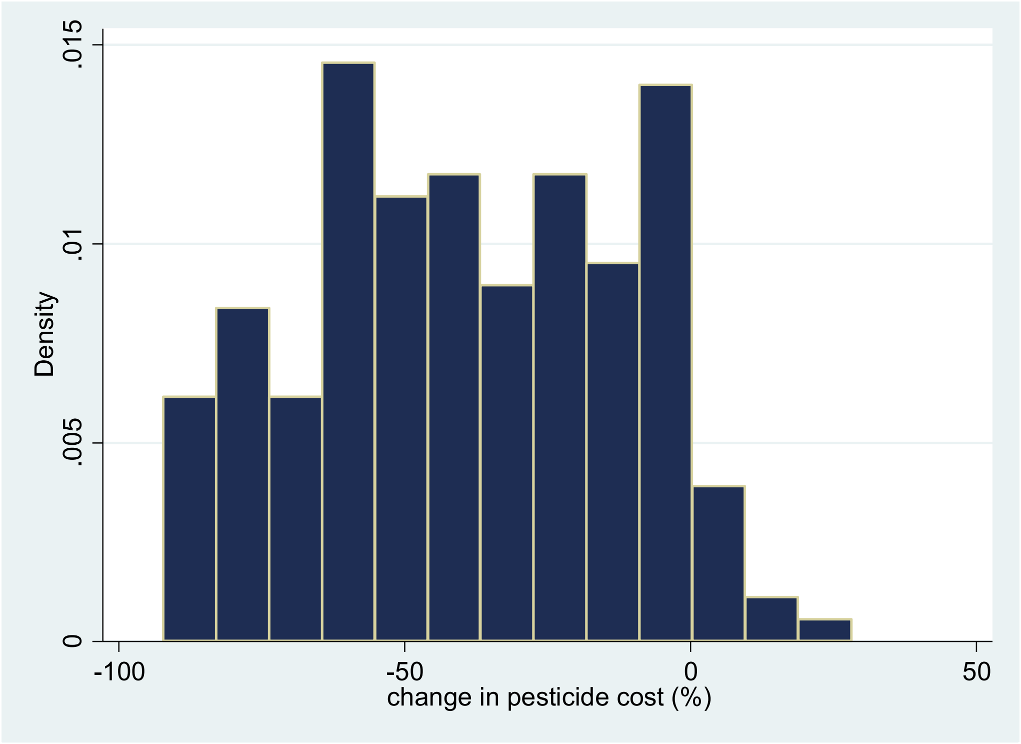

d) Change in total production cost through GM crop adoption (115 observations)

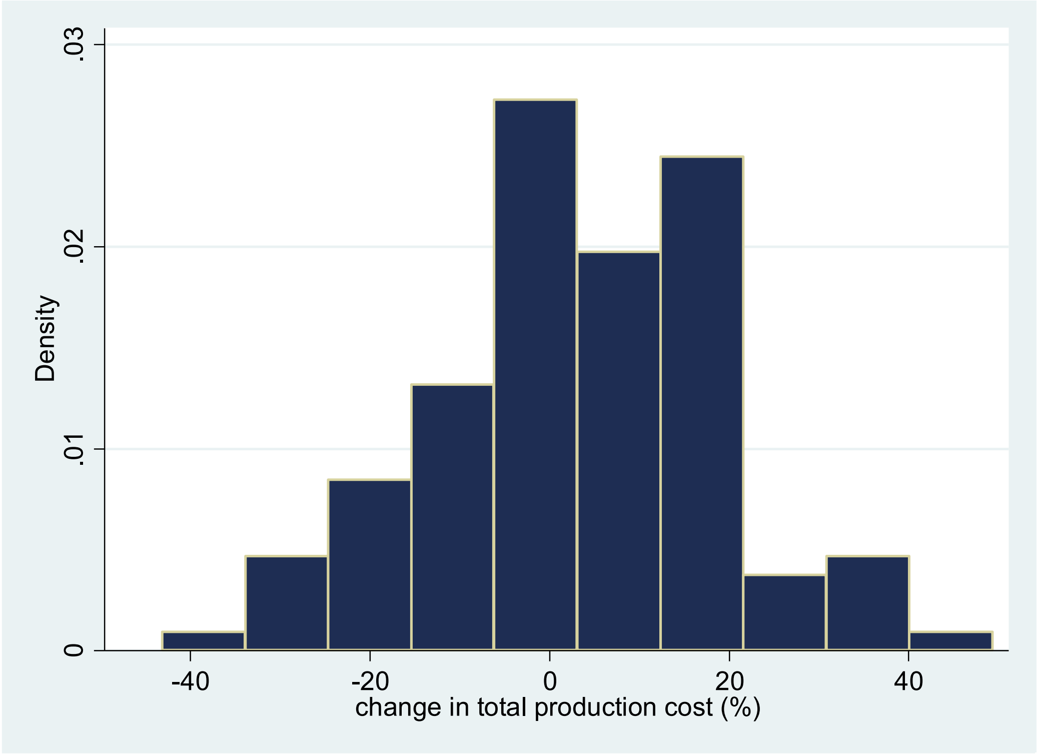

e) Change in farmer profit through GM crop adoption (136 observations)

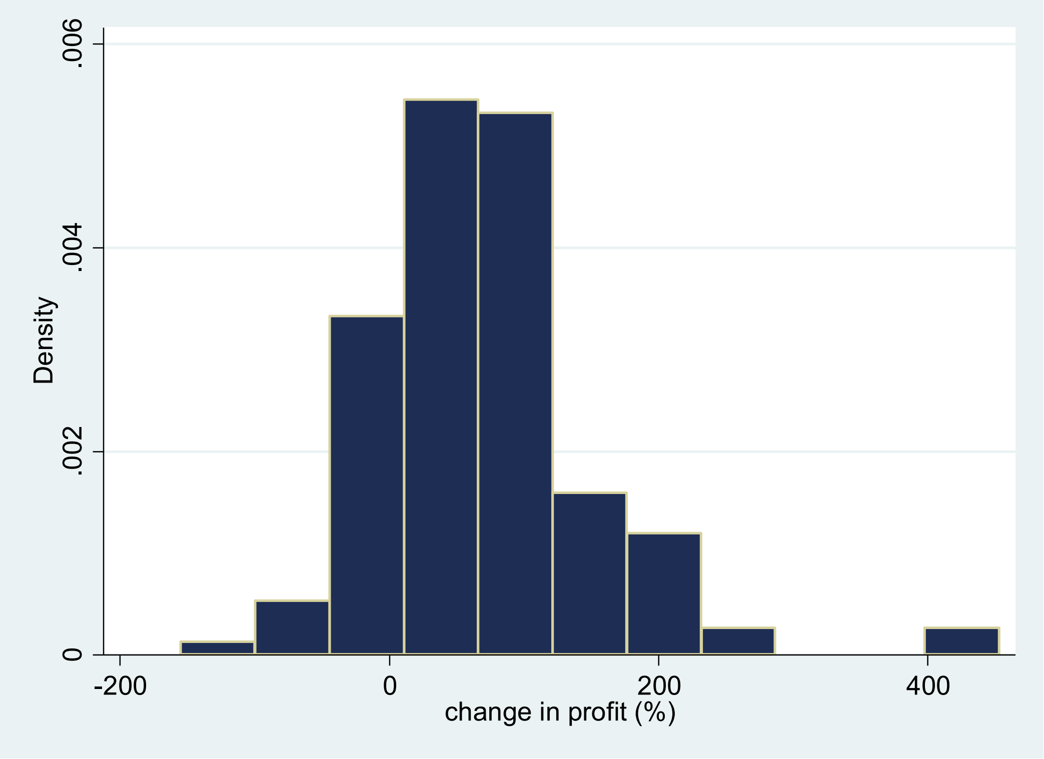

Note: We carried out Cochran's test of heterogeneity for all five outcome variables. The test statistic (Cochran's *Q*), which is distributed as a chi-square statistic, is used to test the null hypothesis that all studies share a common effect size. For the calculation of *Q*, measures of variance are required, which are not available for all original studies. Hence, the test statistics were calculated only with those studies that reported variance measures. For all outcome variables the null hypothesis is rejected. Yield: x^2=3894.6 (p<0.01); pesticide quantity: x^2=1278.7 (p<0.01); pesticide cost: x^2=1930.8 (p<0.01); total production cost: x^2=107.0 (p<0.01); profit: x^2=1959.1 (p<0.01).
