# Supplementary material for: A Meta-Analysis of the Impacts of Genetically Modified Crops
Source: PLoS One. 2014 Nov 3;9(11):e111629. doi: 10.1371/journal.pone.0111629 (PMC4218791; doi:10.1371/journal.pone.0111629)
Supplement: Figure S2 — Impacts of GM crop adoption including only studies published in journals. (PDF) [file pone.0111629.s002.pdf]

Figure S2. Impacts of GM crop adoption including only studies published in journals

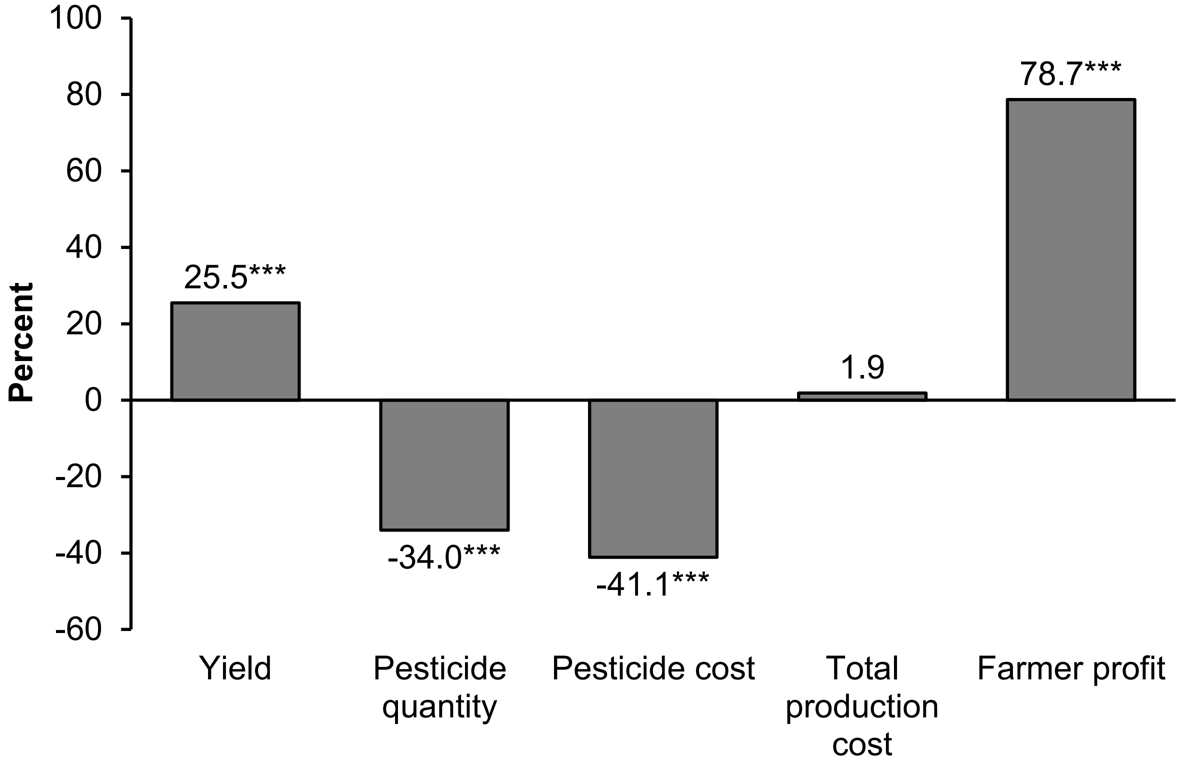

Notes: Average percentage differences between GM and non-GM crops are shown. Results refer to all GM crops, including herbicide-tolerant and insect-resistant traits. The number of observations varies by outcome variable; yield: 317; pesticide quantity: 90; pesticide cost: 126; total production cost: 84; farmer profit: 94. *** indicates statistical significance at the 1% level.
