# Supplementary material for: A Meta-Analysis of the Impacts of Genetically Modified Crops
Source: PLoS One. 2014 Nov 3;9(11):e111629. doi: 10.1371/journal.pone.0111629 (PMC4218791; doi:10.1371/journal.pone.0111629)
Supplement: Figure S3 — Funnel plots for the five outcome variables. (PDF) [file pone.0111629.s003.pdf]

**Figure S3. Funnel plots for the five outcome variables**

*a) Change in yield through GM crop adoption*

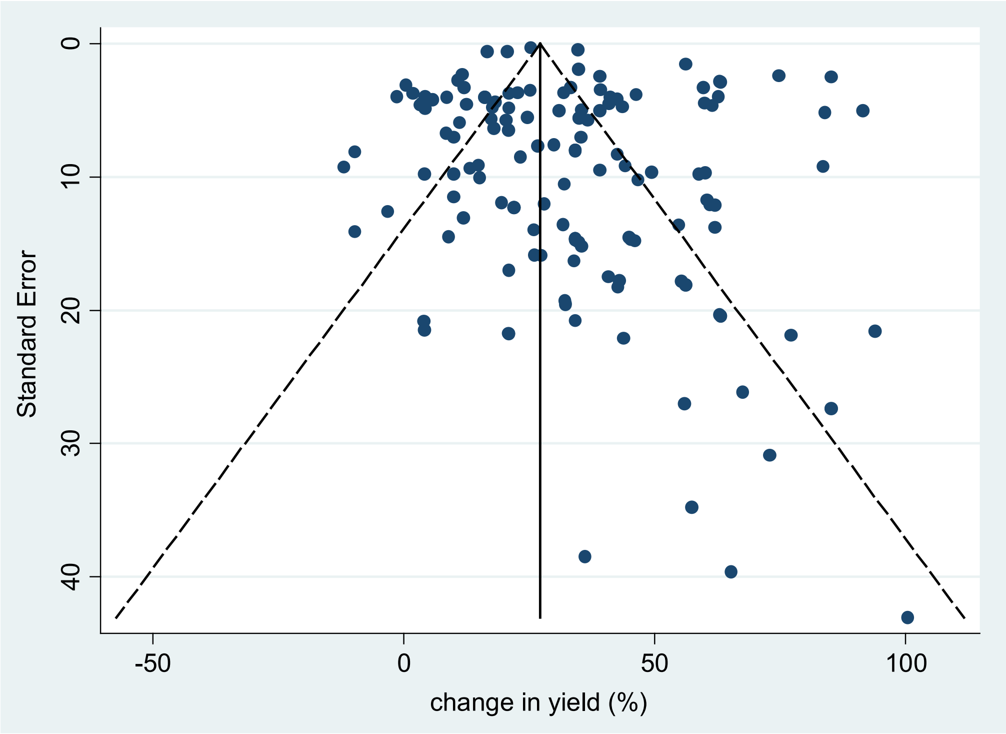


*b) Change in pesticide quantity through GM crop adoption*

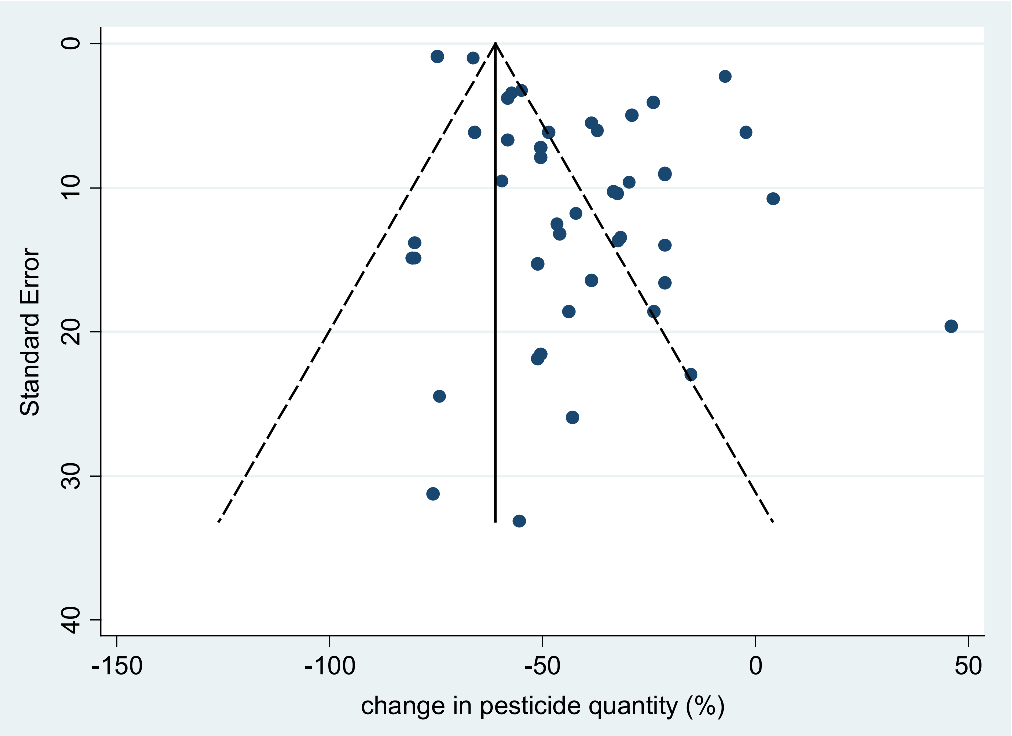

*c) Change in pesticide cost through GM crop adoption*

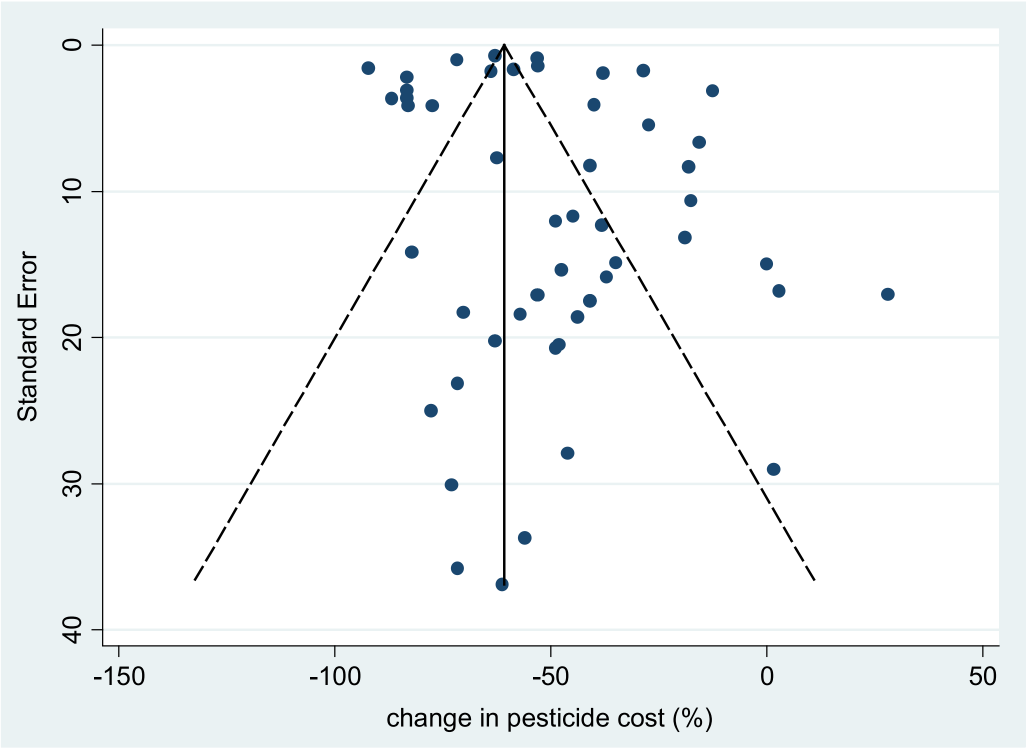


*d) Change in total production cost through GM crop adoption*

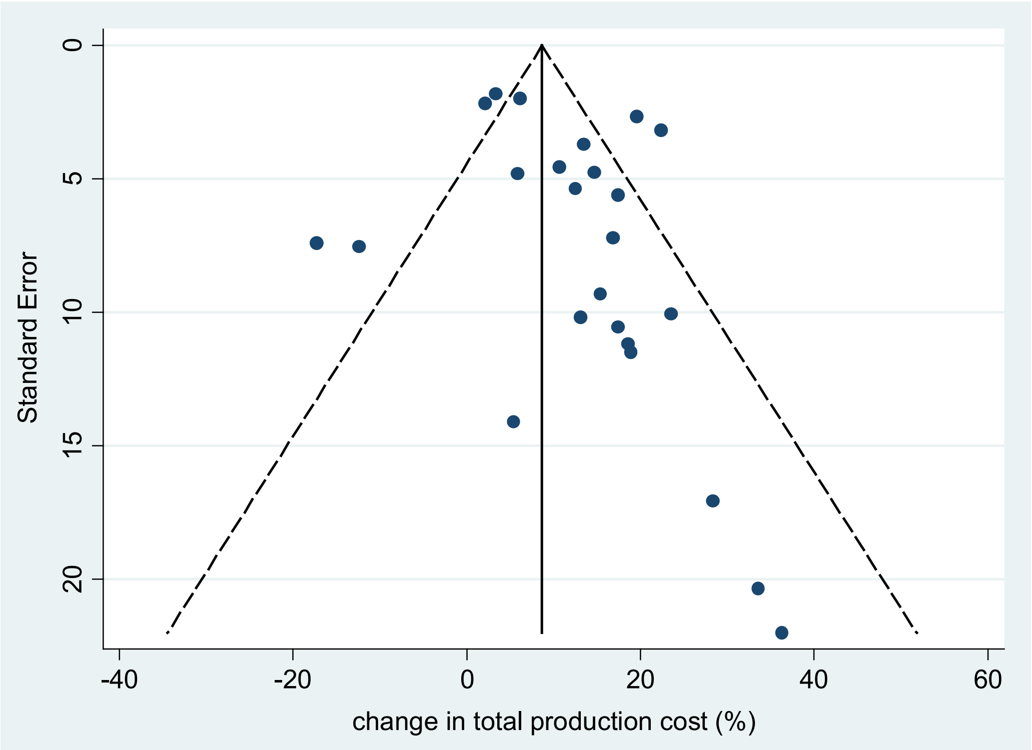

*e) Change in farmer profit through GM crop adoption*

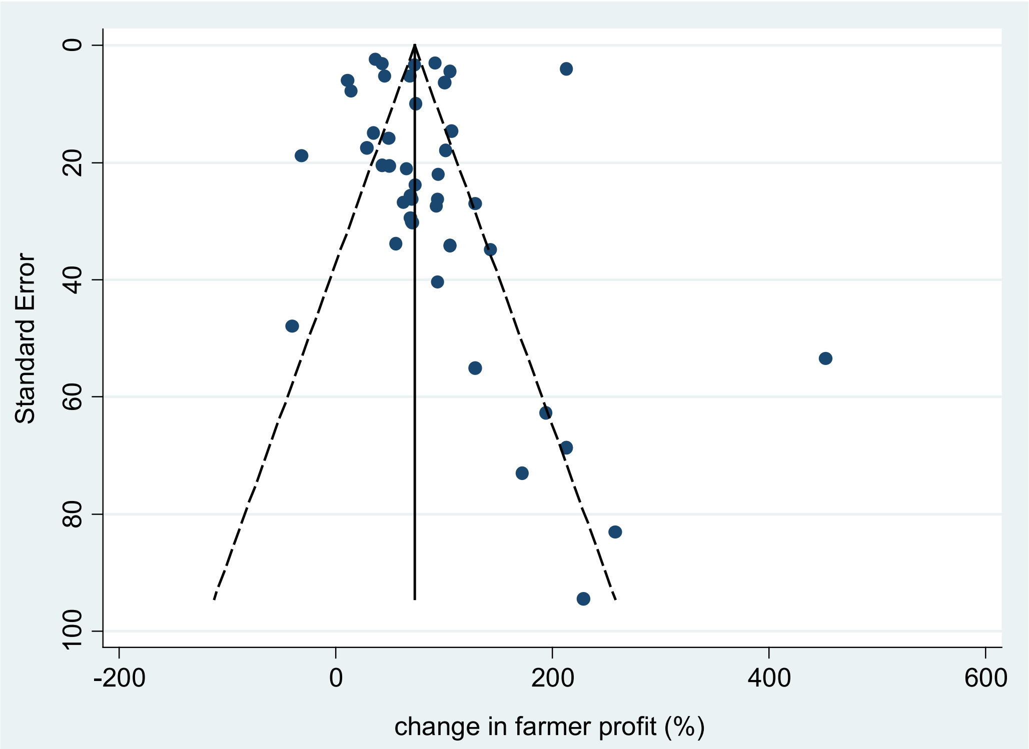


Notes: Funnel plots only include observations from those studies that report measures of variance for the effect sizes. As this is a subset of the total number of observations, the indicated mean values differ from those shown in Figure 2 and Table 2 of the main article. The mean values shown in these funnel plots suggest somewhat stronger effects of GM crops, implying that the main results are rather conservative estimates. The dotted lines are 95% confidence limits.
