# Supplementary material for: A Meta-Analysis of the Impacts of Genetically Modified Crops
Source: PLoS One. 2014 Nov 3;9(11):e111629. doi: 10.1371/journal.pone.0111629 (PMC4218791; doi:10.1371/journal.pone.0111629)
Supplement: Table S1 — List of publications included in the meta-analysis. (PDF) [file pone.0111629.s004.pdf]

Table S1. List of publications included in the meta-analysis

Number	Publication
1	Abdullah, A. An analysis of Bt cotton cultivation in Punjab, Pakistan using the agriculture decision support system (ADSS). *AgBioForum* **13,** 274–287 (2010).
2	Ali, A. & Abdulai, A. The adoption of genetically modified cotton and poverty reduction in Pakistan. *Journal of Agricultural Economics* **61,** 175–192 (2010).
3	Andersen, M. N. Sausse, C. Lacroix, B. Caul, S. & Messean, A. Agricultural studies of GM maize and the field experimental infrastructure of ECOGEN. *Pedobiologia* **51,** 175–184 (2007).
4	Ansingkar, A. S. More, S. S. Bhatade, S. S. Dhuppe, M. V. & Choudhary, L. M. Evaluation of transgenic Bt cotton hybrids in comparison with non-Bt and checks in rainfed condition. *Journal of Soils and Crops* **15,** 338–342 (2005).
5	Badiz Furlaneto, F. d. P. Reco, P. C. Dias Kanthack, R. A. Tsutsui Esperancinil, M. S. & Oliveira Ojima, A. L. R. de. Transgenic versus conventional soybean: estimative of the operational production costs in the Middle Paranapanema, Sao Paulo State. *Ciencia e Agrotecnologia* **32,** 1935–1940 (2008).
6	Banerjee, S. B. & Martin, S. W. An estimation of producer returns from Bt cotton with varying refuge sizes. *Crop Protection* **27,** 1003–1008 (2008).
7	Barry, B. D. *et al.* Performance of transgenic corn hybrids in Missouri for insect control and yield. *Journal of Economic Entomology* **93,** 993–999 (2000).
8	Barwale, R. B. Gadwal, V. R. Zehr, U. & Zehr, B. Prospects for Bt cotton technology in India. *AgBioForum* **7,** 23–26 (2004).
9	Benbrook, C. *Evidence of the Magnitude and Consequences of the Roundup Ready Soybean Yield Drag from University-Based Varietal Trials in 1998*. (Ag Biotech InfoNet, Idaho, 1999).
10	Bennett, R. Buthelezi, T. J. Ismael, Y. & Morse, S. Bt cotton, pesticides, labour and health - A case study of smallholder farmers in the Makhathini Flats, Republic of South Africa. *Outlook on Agriculture* **32,** 123–128 (2003).
11	Bennett, R. M. Ismael, Y. Kambhampati, U. & Morse, S. Economic impact of genetically modified cotton in India. *AgBioForum* **7,** 96–100 (2004).
12	Bennett, R. Ismael, Y. & Morse, S. Explaining contradictory evidence regarding impacts of genetically modified crops in developing countries: varietal performance of transgenic cotton in India. *Journal of Agricultural Science* **143,** 35–42 (2005).
13	Bennett, R. Ismael, Y. Morse, S. & Shankar, B. Reductions in insecticide use from adoption of Bt cotton in South Africa: impacts on economic performance and toxic load to the environment. *Journal of Agricultural Science* **142,** 665–674 (2004).
14	Bennett, R. Kambhampati, U. Morse, S. & Ismael, Y. Farm-level economic performance of genetically modified cotton in Maharashtra, India. *Review of Agricultural Economics* **28,** 59–71 (2006).
15	Bennett, R. Morse, S. & Ismael, Y. The economic impact of genetically modified cotton on South African smallholders: yield, profit and health effects. *Journal of Development Studies* **42,** 662–677 (2006).
16	Beyers, L. Ismael, Y. & Piesse, J. Can GM-technologies help the poor? The impact of Bt cotton in Makhathini Flats, Kwazulu-Natal. *World Development* **31,** 717–732 (2003).
17	Beyers, L. & Thirtle, C. G. *Can GM-Technologies Help African Smallholders? The Impact of Bt Cotton in the Makhathini Flats of Kwazulu-Natal.* International Association of Agricultural Economists 2003 Annual Meeting (Durban, South Africa, 2003).
18	Bheemanna, M. Patil, B. V. Hanchinal, S. G. Hosamani, A. C. & Bansi, A. B. Comparative performance and economics of Bollgard-II Bt cotton under irrigated conditions. *Journal of Cotton Research and Development* **22,** 118–121 (2008).
19	Brethour, C. Mussell, A. Mayer, H. & Martin Larry. *Agronomic, Economic and Environmental Impacts of the Commercial Cultivation of Glyphosate Tolerant Soybeans in Ontario* (George Morris Centre, Guelph, 2002).
20	Brookes, G. The farm-level impact of herbicide-tolerant soybeans in Romania. *AgBioForum* **8,** 235–241 (2005).
21	Cattaneo, M. G. *et al.* Farm-scale evaluation of the impacts of transgenic cotton on biodiversity, pesticide use, and yield. *Proceedings of the National Academy of Sciences* **103,** 7571–7576 (2006).
22	Ceddia, M. G. Gomez-Barbero, M. & Rodriguez-Cerezo, E. An ex-ante evaluation of the economic impact of Bt cotton adoption by Spanish farmers facing the EU cotton sector reform, *AgBioForum* **11,** 82–92 (2008).
23	Crost, B. Shankar, B. Bennett, R. & Morse, S. Bias from farmer self-selection in genetically modified crop productivity estimates: evidence from Indian data. *Journal of Agricultural Economics* **58,** 24–36 (2007).
24	Dev, S. M. & Rao, N. C. *Socioeconomic Impact of Bt Cotton* (Centre for Economic and Social Studies, Hyderabad, 2007).

25	Dhillon, M. K. Pampapathy, G. Wadaskar, R. M. & Sharma, H. C. Impact of Bt transgenic cottons and insecticides on target and non-target insect pests, natural enemies and seedcotton yield in India. *Indian Journal of Agricultural Sciences* **82** (2012).
26	Diaz-Osorio, J. Herrera R. Valderrama J. & Llanos-Ascencio J. L. Potential changes in the competitiveness of maize growers in Central Chile through the use of transgenic seed (Bt and RR). *Spanish Journal of Agricultural Research* **2** (2004).
27	Dong, H. Li, W. Tang, W. & Zhang, D. Development of hybrid Bt cotton in China - A successful integration of transgenic technology and conventional techniques. *Current Science* **86,** 778–782 (2004).
28	Doyle, B. Reeve I. & Barclay, E. The Performance of Ingard Cotton in Australia during the 2000/2001 Season. Institute for Rural Futures, University of New England, Armidale, New South Wales, Australia (2002).
29	Doyle, B. Reeve I. & Bock K. The Performance of Ingard Cotton in Australia during the 2001/2002 Season. Institute for Rural Futures, University of New England, Armidale, New South Wales, Australia (2002).
30	Duffy, M. &. E. M. Does planting GMO seed boost farmers' profits? *Leopold Letter*, 1999.
31	Duffy, M. *Who Benefits from Biotechnology?* Presented at the American Seed Trade Association Meeting (Chicago, IL, 2001).
32	Falck-Zepeda, J. T. G. a. N. R. Rent creation and distribution from biotechnology innovations: the case of Bt cotton and herbicide-tolerant soybeans in 1997. *Agribusiness* **16,** 21–32 (2000).
33	Fernandez-Cornejo, J. & Li, J. *The Impacts of Adopting Genetically Engineered Crops in the USA: The Case of Bt Corn.* Presented at the American Agricultural Economics Association Annual Meeting (Providence, Rhode Island, 2005).
34	Fernandez-Cornejo, J. & Wechsler, S. *Revisiting the Impact of Bt Corn Adoption by U.S. Farmers.* Agricultural and Applied Economics Association's AAEA and NAREA Joint Annual Meeting (Pittsburgh, PA, 2011).
35	Fernandez-Cornejo, J. & Wechsler, S. J. Revisiting the impact of Bt corn adoption by U.S. farmers. *Agricultural and Resource Economics Review* **41,** 377–390 (2012).
36	Fernandez-Cornejo, J. & Wechsler, S. *Fifteen Years Later: Examining the Adoption of Bt Corn Varieties by US Farmers.* Presented at the Agricultural and Applied Economics Association's AAEA Annual Meeting (Seattle, VA, 2012).
37	Fok, M. A. C. Gouse M. Hofs J.-L. & Kirsten J. Contextual appraisal of GM cotton diffusion in South Africa. *Life Sciences International Journal* **1,** 468–482 (2007).
38	Gandhi, V. P. & Namboodiri, N. V. The Adoption and Economics of Bt Cotton in India: Preliminary Results from a Study. Indian Institute of Management, Ahmedabad, 2006.
39	Gaurav, S. & Mishra, S. To Bt or not to Bt? Risk and Uncertainty Considerations in Technology Assessment. Indira Gandhi Institute of Development Research, Mumbai, 2012.
40	Gómez-Barbero, M., Berbel, J. & Rodríguez-Cerezo, E. Adoption and performance of the first GM crop introduced in EU agriculture: Bt maize in Spain. Joint Research Center, Seville, 2008.
41	Gouse, M. GM Maize as subsistence crop: the South African smallholder experience. *AgBioForum* **15,** 163–174 (2012).
42	Gouse, M. Gouse, M. Piesse, J. & Thirtle, C. G. Output and labour effects of GM maize and minimum tillage in a communal area of Kwa-Zulu-Natal. *Journal of Development Perspectives* **2,** 71–86 (2006).
43	Gouse, M. Kirsten, J. F. & Jenkins, L. Bt cotton in South Africa: Adoption and the impact on farm incomes amongst small-scale and large scale farmers. *Agrekon* **42,** 15–29 (2003).
44	Gouse, M. Piesse, J. Thirtle, C. & Poulton, C. Assessing the performance of GM maize amongst smallholders in KwaZulu-Natal, South Africa. *AgBioForum* **12,** 78–89 (2009).
45	Gouse, M. Pray, C. E. Kirsten, J. & Schimmelpfennig, D. A GM subsistence crop in Africa: the case of Bt white maize in South Africa. *International Journal of Biotechnology* **7,** 84–94 (2005).
46	Gouse, M. Pray, C. Schimmelpfennig, D. & Kirsten, J. Three seasons of subsistence insect-resistant maize in South Africa: have smallholders benefited? *AgBioForum* **9,** 15–22 (2006).
47	Government of Andhra Pradesh. Performance Report of Bt Cotton in Andhra Pradesh. Report of State Department of Agriculture, Hyderabad, 2003.
48	Gruere, G. P. & Sun, Y. Measuring the Contribution of Bt Cotton Adoption to India's Cotton Yields Leap. IFPRI Discussion Paper 01170, International Food Policy Research Institute, Washington, DC, 2012.
49	Gulden, R. H. Sikkema, P. H. Hamill, A. S. Tardif, F. & Swanton, C. J. Conventional vs. glyphosate-resistant cropping systems in Ontario: weed control, diversity, and yield. *Weed Science* **57,** 665–672 (2009).
50	Hategekimana, B. Genetically Modified Grain Corn and Soybean in Quebec and Ontario in 2000 and 2001. Agriculture and Rural Working Paper Series 54. Statistics Canada, 2002.

51	Heatherly, L. G. Elmore, C. D. & Spurlock, S. R. Weed management systems for conventional and glyphosate-resistant soybean with and without irrigation. *Agronomy Journal* **94,** 1419–1428 (2002).
52	Hofs, J. L. Hau, B. Marais, D. & Fok, M. Boll distribution patterns in Bt and non-Bt cotton cultivars: II. Study on small-scale farming systems in South Africa. *Field Crops Research* **98,** 210–215 (2006).
53	Hofs, J.-L. Fok, M. & Vaissayre, M. Impact of Bt cotton adoption on pesticide use by smallholders: A 2-year survey in Makhathini Flats (South Africa). *Crop Protection* **25,** 984–988 (2006).
54	Hu, R. *et al.* Reforming intellectual property rights and the Bt cotton seed industry in China: who benefits from policy reform? *Research Policy* **38,** 793–801 (2009).
55	Huang, J. K. Rozelle, S. Pray, C. & Wang, Q. F. Plant biotechnology in China. *Science* **295,** 674–677 (2002).
56	Huang, J. Hu, R. & Fan, C. Bt cotton benefits, costs, and impacts in China. *AgBioForum* **5,** 153–166 (2002).
57	Huang, J. Hu, R. Pray, C. Qiao, F. & Rozelle, S. Biotechnology as an alternative to chemical pesticides: a case study of Bt cotton in China. *Agricultural Economics* **29,** 55–67 (2003).
58	Huang, J. Hu, R. Rozelle, S. Qiao, F. & Pray, C. Small holders, transgenic varieties, and production efficiency: the case of cotton farmers in China. Dept. of Agricultural & Resource Economics Working Paper 01-015, University of California, Davis 2001.
59	Huang, J. Hu, R. Rozelle, S. Qiao, F. & Pray, C. E. Transgenic varieties and productivity of smallholder cotton farmers in China. *Australian Journal of Agricultural and Resource Economics* **46,** 367–387 (2002).
60	IMRB International. Socio Economic Benefits of Bollgard and Product Satisfaction (in India). Indian Market Research Bureau (IMRB) International, Mumbai (2005).
61	Ismael, Y. Bennett, R. & Morse, S. Benefits from Bt cotton use by smallholder farmers in South Africa. *AgBioForum* **5,** 1–5 (2002).
62	Ismael, Y. Bennett, R. & Morse, S. *Biotechnology in Africa: The Adoption and Economic Impacts of Bt Cotton in the Makhathini Flats, Republic of South Africa.* Paper presented for AfricaBio Conference: Biotechnology Conference for Sub-Saharan Africa (Johannesburg, South Africa, 2001).
63	Janaki, P. & Raja, D. Performance, benefits and impact of Bt cotton production in Salem, India. *Agriculture Update* **4,** 171–173 (2009).
64	Kapadiya, H. J. Butani, A. M. Khanpara, M. D. & Nariya, J. N. Farmers' participation in front line demonstration (FLD) for Bt cotton in Saurashtra region of Gujarat. *Journal of Cotton Research and Development* **26,** 137–141 (2012).
65	Kathage, J. & Qaim, M. Economic impacts and impact dynamics of Bt (Bacillus thuringiensis) cotton in India. *Proceedings of the National Academy of Sciences* **109,** 11652–11656 (2012).
66	Keetch, D. P. Ngqaka, A. Akanbi, R. & Mahlanga, P. Bt maize for small scale farmers: a case study. *African Journal of Food, Agriculture, Nutrition and Development* **4,** 1505–1509 (2005).
67	Kiresur, V. R. & Ichangi, M. Socio-economic impact of Bt cotton—a case study of Karnataka. *Agricultural Economics Research Review* **24** (2011).
68	Klotz-Ingram, C. a. Farm-level production effects related to the adoption of genetically modified cotton for pest management. *AgBioForum* **2,** 73–84 (1999).
69	Kouser, S. & Qaim, M. Impact of Bt cotton on pesticide poisoning in smallholder agriculture: a panel data analysis. *Ecological Economics* **70,** 2105–2113 (2011).
70	Kouser, S. & Qaim, M. *Valuing financial, health, and environmental benefits of Bt cotton in Pakistan.* Presented at the International Association of Agricultural Economists (IAAE) Triennial Conference (Foz do Iguaçu, Brazil, 18-24 August, 2012).
71	Krishna, V. V. & Qaim, M. Bt cotton and sustainability of pesticide reductions in India. *Agricultural Systems* **107,** 47–55 (2012).
72	Lalitha, N. R. B. &. V. P. in *Biotechnology and agricultural development: Transgenic cotton, rural institutions and resource-poor farmers,* edited by R. Tripp (Routledge, Abingdon, Oxon, 2009), pp. 135–167.
73	Lin, W. Price, G. K. & Fernandez-Cornejo, J. Estimating farm-level effects of adopting herbicide-tolerant soybeans. *Oil Crops Situation and Outlook* October 2001, 25–34 (United States Department of Agriculture, Washington, DC, 2001).
74	Loganathan, R. Balasubramanian, R. Mani, K. & Gurunathan, S. Productivity and profitability impact of genetically modified crops – an economic analysis of Bt cotton cultivation in Tamil Nadu. *Agricultural Economics Research Review* **22,** 331–340 (2009).
75	Ma, B. L. Meloche, F. & Wei, L. Agronomic assessment of Bt trait and seed or soil-applied insecticides on the control of corn rootworm and yield. *Field Crops Research* **111,** 189–196 (2009).
76	Ma, B. L. & Subedi, K. D. Development, yield, grain moisture and nitrogen uptake of Bt corn hybrids and their

	conventional near-isolines. *Field Crops Research* **93,** 199–211 (2005).
77	Mal, P. Manjunatha, A. V. Bauer, S. & Ahmed, M. N. Technical efficiency and environmental impact of Bt cotton and non-Bt cotton in North India. *AgBioForum* **14,** 164–170 (2011).
78	McBride, W. D. & Brooks, N. Survey evidence on producer use and costs of genetically modified seed. *Agribusiness* **16,** 6–20 (2000).
79	McBride, W. D. & El-Osta, H. S. Impacts of the adoption of genetically engineered crops on farm financial performance. *Journal of Agricultural and Applied Economics* **34,** 175–191 (2002).
80	Ministry of Agriculture of the Czech Republic. Experience with Bt maize cultivation in the Czech Republic 2005–2009 (Ministry of Agriculture of the Czech Republic, Prague, 2010).
81	Morse, S. Bennett, R. & Ismael, Y. Why Bt cotton pays for small-scale producers in South Africa. *Nature Biotechnology* **22,** 379–380 (2004).
82	Morse, S. Bennett, R. M. & Ismael, Y. Genetically modified insect resistance in cotton: some farm level economic impacts in India. *Crop Protection* **24,** 433–440 (2005).
83	Morse, S. Bennett, R. & Ismael, Y. Environmental impact of genetically modified cotton in South Africa. *Agriculture, Ecosystems & Environment* **117,** 277–289 (2006).
84	Morse, S. Bennett, R. & Ismael, Y. Inequality and GM crops: a case-study of Bt cotton in India. *AgBioForum* **10,** 44–50 (2007).
85	Morse, S. Bennett, R. & Ismael, Y. Isolating the 'farmer' effect as a component of the advantage of growing genetically modified varieties in developing countries: a Bt cotton case study from Jalgaon, India. *Journal of Agricultural Science* **145,** 491–500 (2007).
86	Muhammad, S. A. A comparative farm level cultivation of conventional and Bt cotton. *Scientific Papers. Series A. Agronomy* **55,** 207–211 (2012).
87	Mungai, N. W. Motavalli, P. P. Nelson, K. A. & Kremer, R. J. Differences in yields, residue composition and N mineralization dynamics of Bt and non-Bt maize. *Nutrient Cycling in Agroecosystems* **73,** 101–109 (2005).
88	Mutuc, M. E. M. Rejesus, R. M. Pan, S. & Yorobe Jr, J. M. Impact assessment of Bt corn adoption in the Philippines. *Journal of Agricultural and Applied Economics* **44,** 117–135 (2012).
89	Mutuc, M. E. Rejesus, R. M. & Yorobe, J. M. JR. Yields, insecticide productivity, and Bt corn: evidence from damage abatement models in the Philippines. *AgBioForum* **14,** 35–46 (2011).
90	Mutuc, M. Rejesus, R. M. & Yorobe, J. M. Which farmers benefit the most from Bt corn adoption? Estimating heterogeneity effects in the Philippines. *Agricultural Economics* **44** (2013).
91	Naik, G. Qaim M. & Zilberman D. Bt cotton controversy – some paradoxes explained. *Economic and Political Weekly* **40,** 1514–1517 (2005).
92	Narayanamoorthy, A. & Kalamkar, S. S. Is Bt cotton cultivation economically viable for Indian farmers? An empirical analysis. *Economic and Political Weekly* **41,** 2716–2724 (2006).
93	Nazli, H. Sarker, R. Meilke, K. D. & Orden, D. *Economic Performance of Bt Cotton Varieties in Pakistan.* Presented at the Agricultural and Applied Economics Association 2010 Annual Meeting (Denver, Colorado, 2010).
94	Nolte, S. A. & Young, B. G. Efficacy and economic return on investment for conventional and herbicide-resistant soybean (Glycine max). *Weed Technology* **16,** 388–395 (2002).
95	Orphal, J. Comparative Analysis of the Economics of Bt and non-Bt Cotton Production. Institute of Economics in Horticulture, Universität Hannover, Germany (2013).
96	Owen, M. D. K. *et al.* Comparisons of genetically modified and non-genetically modified soybean cultivars and weed management systems. *Crop Science* **50,** 2597–2604 (2010).
97	Pemsl, D. Waibel, H. & Orphal, J. A methodology to assess the profitability of Bt-cotton: case study results from the state of Karnataka, India. *Crop Protection* **23,** 1249–1257 (2004).
98	Penna, J. A. & Lema, D. in *The Economic and Environmental Impacts of Agbiotech: A Global Perspective,* edited by N. Kalaitzandonakes (Kluwer Academic/Plenum, New York, Dordrecht and London, 2003), pp. 203–221.
99	Peshin, R. Dhawan, A. K. Vatta, K. & Singh, K. Attributes and socio-economic dynamics of adopting Bt cotton. *Economic and Political Weekly,* 73–80 (2007).
100	Pray, C. Ma, D. Huang, J. & Qiao, F. Impact of Bt cotton in China. *World Development* **29,** 813–825 (2001).
101	Price, G. Lin, W. Falck-Zepeda, J. & Fernandez-Cornejo, J. Size and Distribution of Market Benefits From Adopting Biotech Crops. USDA Technical Bulletin 1906, United States Department of Agriculture, Washington, DC, 2003.
102	Qaim, M. Bt cotton in India: Field trial results and economic projections. *World Development* **31,** 2115–2127 (2003).

103	Qaim, M. Cap, E. J. & Janvry, A. de. Agronomics and sustainability of transgenic cotton in Argentina. *AgBioForum* **6,** 41–47 (2003).
104	Qaim, M. & de Janvry, A. Genetically modified crops, corporate pricing strategies, and farmers' adoption: the case of Bt cotton in Argentina. *American Journal of Agricultural Economics* **85,** 814–828 (2003).
105	Qaim, M. & de Janvry, A. Bt cotton and pesticide use in Argentina: economic and environmental effects. *Environment and Development Economics* **10,** 179–200 (2005).
106	Qaim, M. & Matuschke, I. Impacts of genetically modified crops in developing countries: a survey. *Quarterly Journal of International Agriculture* **44,** 207–227 (2005).
107	Qaim, M. Subramanian, A. Naik, G. & Zilberman, D. Adoption of Bt cotton and impact variability: insights from India. *Review of Agricultural Economics* **28,** 48–58 (2006).
108	Qaim, M. & Traxler, G. Roundup Ready soybeans in Argentina: farm level and aggregate welfare effects. *Agricultural Economics* **32,** 73–86 (2005).
109	Qayum, A. & Sakkhari, K. Bt Cotton in Andhra Pradesh – 3 Year Assessment. The First Sustained Independent Scientific Study of Bt Cotton in India. Deccan Development Society, Hyderabad, 2005.
110	Qayum, A. & Sakkhari, K. Did Bt Cotton Save Farmers in Warangal? A Season Long Impact Study of Bt Cotton - Kharif 2002. Deccan Development Society, Hyderabad, 2003.
111	Ramasundaram, P. Vennila, S. & Ingle, R. Bt cotton performance and constraints in central India. *Outlook on Agriculture* **36,** 175–180 (2007).
112	Rao, N. C. & Dev, S. M. Socio-economic impact of transgenic cotton. *Agricultural Economics Research Review* **22,** 461–470 (2009).
113	Reddy, K. N. & Whiting, K. Weed control and economic comparisons of glyphosate-resistant, sulfonylurea-tolerant, and conventional soybean (Glycine max) systems. *Weed Technology* **14,** 204–211 (2000).
114	Sadashivappa, P. & Qaim, M. Bt cotton in India: development of benefits and the role of government seed price interventions. *AgBioForum* **12,** 172–183 (2009).
115	Sahai, S. &. Rahman S. Performance of Bt cotton: data from first commercial crop. *Economic and Political Weekly* **38,** 3139–3141 (2003).
116	Sahai, S. & Rahman, S. Bt-Cotton performance 2003-2004: fields swamped with illegal variants. *Economic and Political Weekly* **39,** 2673–2674 (2004).
117	Sanglestsawai, S. Rejesus, R. M. & Yorobe Jr, J. M. *Production Risk, Farmer Welfare, and Bt Corn in the Philippines.* Selected Paper prepared for presentation at the AAEA Annual Meeting (Seattle, WA, 2012).
118	Sankula, S. Quantification of the impacts on US agriculture of biotechnology-derived crops planted in 2005. National Center for Food and Agricultural Policy, 2006.
119	Sankula, S. Marmon, G. & Blumenthal, E. Biotechnology-derived crops planted in 2004: impacts on US agriculture. National Center for Food and Agricultural Policy, Washington, DC, 2005.
120	Sarang, D. H. Bhatade, S. S. & Deosarkar, D. B. Evaluation of some new Bt cotton hybrids for seed cotton yield and fiber quality traits under rainfed conditions. *Journal of Cotton Research and Development* **24,** 149–154 (2010).
121	Sexton, S. E. & Zilberman, D. How Agricultural Biotechnology Boosts Food Supply and Accommodates Biofuels. NBER Working Paper Series 16699, National Bureau of Economic Research, 2011.
122	Shankar, B. Bennett, R. & Morse, S. Production risk, pesticide use and GM crop technology in South Africa. *Applied Economics* **40,** 2489–2500 (2008).
123	Sharma, H. C. & Pampapathy, G. Influence of transgenic cotton on the relative abundance and damage by target and non-target insect pests under different protection regimes in India. *Crop Protection* **25,** 800–813 (2006).
124	Shi, G. Chavas, J.-P. & Lauer, J. Commercialized transgenic traits, maize productivity and yield risk. *Nature Biotechnology* **31,** 111–114 (2013).
125	Shi, G. Chavas, J.-P. Lauer, J. & Nolan, E. An analysis of selectivity in the productivity evaluation of biotechnology: an application to corn. *American Journal of Agricultural Economics* **95,** 1–16 (2013).
126	Shiva, V. Toxic genes and toxic papers: IFPRI covering up the link between Bt Cotton and farmers suicides. Research Foundation for Science, Technology and Ecology, New Delhi, 2008.
127	Singh, D. Pandey, R. & Kumar, V. Performance of Bt and non Bt cotton hybrids at wider spacing in north western plain zones. *Journal of Cotton Research and Development* **25,** 217–220 (2011).
128	Skevas, T. Fevereiro, P. & Wesseler, J. Coexistence regulations and agriculture production: a case study of five Bt maize producers in Portugal. *Ecological Economics* **69,** 2402–2408 (2010).
129	Speese, J. *et al.* Efficacy and economics of fresh-market Bt transgenic sweet corn in Virginia. *Crop Protection* **24,** 57–64 (2005).

130	Stone, G. D. Field versus Farm in Warangal: Bt cotton, higher yields, and larger questions. *World Development* **39,** 387–398 (2011).
131	Subramanian, A. & Qaim, M. Village-wide effects of agricultural biotechnology: the case of Bt cotton in India. *World Development* **37,** 256–267 (2009).
132	Subramanian, A. & Qaim, M. *Rural poverty and employment effects of Bt cotton in India*. Presented at International Association of Agricultural Economists (IAAE) Conference (Beijing, China, 2009).
133	Sydorovych, O. & Marra, M. C. A genetically engineered crop's impact on pesticide use: a revealed-preference index approach. *Journal of Agricultural and Resource Economics* **32,** 476–491 (2007).
134	Thirtle, C. Beyers, L. Ismael, Y. & Piesse, J. Can GM-technologies help the poor? The impact of Bt cotton in Makhathini Flats, KwaZulu-Natal. *World Development* **31,** 717–732 (2003).
135	Uys, T. J. E. The Adoption of Plant Biotechnology by Commercial Cotton Producers in South Africa. Master's Thesis. University of Wales, Cardiff, 2004.
136	Vado, L. & Goodwin, B. K. *Analyzing the Effects of Weather and Biotechnology Adoption on Corn Yields and Crop Insurance Performance in the US Corn Belt.* Selected Paper prepared for presentation at the Agricultural & Applied Economics Association's 2010 AAEA, CAES & WAEA Joint Annual Meeting (Denver, CO, 2010).
137	Visawadia, H. Fadadu, A. & Tarpara, V. A comparative analysis of production and marketing of Bt cotton and hybrid cotton in Saurashtra Region of Gujarat State. *Agricultural Economics Research Review* **19,** 293–300 (2006).
138	Vitale, J. Boyer, T. Uaiene, R. & Sanders, J. H. The economic impacts of introducing Bt technology in smallholder cotton production systems of West Africa: a case study from Mali. *AgBioForum* **10,** 71–84 (2007).
139	Vitale, J. Glick, H. Greenplate, J. Abdeennadher, M. & Traore, O. Second-generation Bt cotton field trials in Burkina Faso: Analyzing the potential benefits to West African farmers, *Crop Science* **48,** 1958–1966 (2008).
140	Vitale, J. D. Vognan, G. Ouattarra, M. & Traore, O. The commercial application of GMO crops in Africa: Burkina Faso's decade of experience with Bt cotton. *AgBioForum* **13,** 320–332 (2010).
141	Vries, B. D. de & Fehr, W. R. Impact of the MON89788 event for glyphosate tolerance on agronomic and seed traits of soybean. *Crop Science* **51,** 1023–1027 (2011).
142	Wang, S. Just, D. & Pinstrup-Andersen, P. Bt-cotton and secondary pests. *International Journal of Biotechnology* **10,** 113–121 (2008).
143	Wang, S. Just, D. R. & Pinstrup-Andersen, P. *Tarnishing silver bullets: Bt technology adoption, bounded rationality and the outbreak of secondary pest infestations in China.* Selected Paper prepared for presentation at the American Agricultural Economics Association Annual Meeting (Long Beach, CA, 2006).
144	Wossink, A. & Denaux, Z. Environmental and cost efficiency of pesticide use in transgenic and conventional cotton production. *Agricultural Systems* **90,** 312–328 (2006).
145	Xu, J. You Z. Wang W. & Yang Y. Economic analysis of Bt cotton planting in Jiangsu. *Journal of Yangzhou University (Agricultural and Life Science Edition)* **25,** 65–69 (2004).
146	Yorobe, J. JR. & Quicoy, C. Economic impact of Bt corn in the Philippines. *The Philippine Agricultural Scientist* **89,** 258–267 (2006).
147	Zambrano, P. Fonseca L.A. Cardona I. & Magalhaes E. in *Biotechnology and agricultural development: Transgenic cotton, rural institutions and resource-poor farmers,* edited by R. Tripp (Routledge, Abingdon, Oxon, 2009), pp. 168–199.
