# Supplementary material for: A Meta-Analysis of the Impacts of Genetically Modified Crops
Source: PLoS One. 2014 Nov 3;9(11):e111629. doi: 10.1371/journal.pone.0111629 (PMC4218791; doi:10.1371/journal.pone.0111629)
Supplement: Table S2 — Distribution of study descriptor dummy variables for different outcomes. (PDF) [file pone.0111629.s005.pdf]

Table S2. Distribution of study descriptor dummy variables for different outcomes

Variables	Yield	Pesticide quantity	Pesticide cost	Total cost	Farmer profit
Insect resistance (0/1)	98/353	13/108	48/145	19/96	17/119
Developing country (0/1)	167/287	26/95	49/144	23/92	29/107
Field-trial data (0/1)	363/88	121/0	187/6	105/10	133/3
Industry-funded study (0/1)	430/21	119/2	186/7	111/4	132/4
Regression model result (0/1)	415/36	110/11	190/3	112/3	124/12
Journal publication (0/1)	134/317	31/90	67/126	31/84	42/94
Journal/academic conference (0/1)	106/345	14/107	49/144	29/86	28/108
