# Supplementary material for: A Meta-Analysis of the Impacts of Genetically Modified Crops
Source: PLoS One. 2014 Nov 3;9(11):e111629. doi: 10.1371/journal.pone.0111629 (PMC4218791; doi:10.1371/journal.pone.0111629)
Supplement: Table S3 — Weighted mean impacts of GM crop adoption. (PDF) [file pone.0111629.s006.pdf]

Table S3. Weighted mean impacts of GM crop adoption (%)

Outcome variable	All GM crops	Insect resistance	Herbicide tolerance
Yield	18.66*** (14.01; 23.30)	21.98*** (20.36; 29.93)	6.02* (-0.71; 12.75)
n/*m*	451 / 100	353 / 83	94 / 25
Pesticide quantity	-32.87*** (-43.77; -21.96)	-38.97*** (-49.88; -28.05)	-0.59 (-34.50; 33.31)
n/*m*	121 / 37	108 / 31	13 / 7
Pesticide cost	-38.69*** (-45.90; -31.47)	-39.45*** (-47.85; -31.06)	-36.21*** (-52.38; -20.04)
n/*m*	193 / 57	145 / 45	48 / 15
Total production cost	2.73 (-2.00; 7.45)	3.94* (-0.68; 10.19)	-5.51 (-14.44; 3.40)
n/*m*	115 / 46	96 / 38	19 / 10
Farmer profit	59.37*** (27.88; 90.87)	60.01*** (26.70; 93.31)	56.48 (-58.94; 171.90)
n/*m*	136 / 42	119 / 36	17 / 9

Average percentage differences between GM and non-GM crops are shown with 95% confidence intervals in parentheses. Mean values were calculated using the inverse of the number of impact observations per dataset as weights. *, **, *** indicate statistical significance at the 10%, 5%, and 1% level, respectively. *n* is the number of observations, *m* the number of different primary datasets from which these observations are derived.
