# Supplementary material for: A Meta-Analysis of the Impacts of Genetically Modified Crops
Source: PLoS One. 2014 Nov 3;9(11):e111629. doi: 10.1371/journal.pone.0111629 (PMC4218791; doi:10.1371/journal.pone.0111629)
Supplement: Data S1 — Data used for the meta-analysis. (PDF) [file pone.0111629.s007.pdf]

study_id	crop	d_yield	d_pest	d_pcost	d_totc	d_profit	IR	regression	industry	dev_countr	fieldtrial	jour	jour_conf
8	cotton					49.5	1	1	0	1	0	1	1
8	cotton						1	1	0	1	0	1	1
8	cotton					94.4	1	0	0	1	0	1	1
8	cotton			-37.0			1	0	0	1	0	1	1
8	cotton					43.2	1	1	0	1	0	1	1
8	cotton			0.0			1	0	0	1	0	1	1
8	cotton						1	0	0	1	0	1	1
8	cotton						1	0	0	1	0	1	1
8	cotton	35.5					1	0	0	1	0	1	1
8	cotton	22.7					1	1	0	1	0	1	1
8	cotton						1	0	0	1	0	1	1
8	cotton	21.1					1	1	0	1	0	1	1
8	cotton					70.6	1	0	0	1	0	1	1
8	cotton	40.8					1	0	0	1	0	1	1
8	cotton						1	0	0	1	0	1	1
64	maize	62.5					1	0	0	0	1	1	1
64	maize	12.9					1	0	0	0	1	1	1
64	maize	25.0					1	0	0	0	1	1	1
80	cotton					12.4	1	0	0	0	0	1	1
80	cotton					11.0	1	0	0	0	0	1	1
80	cotton					10.4	1	0	0	0	0	1	1
80	cotton					10.6	1	0	0	0	0	1	1
80	cotton					8.2	1	0	0	0	0	1	1
91	soybean						0	0	0	0	0	1	1
91	soybean		45.7				0	0	0	0	0	1	1
91	soybean		46.0				0	0	0	0	0	1	1
91	soybean						0	0	0	0	0	1	1
91	soybean						0	0	0	0	0	1	1
91	soybean						0	0	0	0	0	1	1
101	maize	5.2					1	0	0	0	1	1	1
101	maize	20.2					1	0	0	0	1	1	1
105	cotton						1	0	0	1	0	1	1
105	cotton			-53.2			1	0	0	1	0	1	1
105	cotton			-63.0			1	0	0	1	0	1	1
105	cotton						1	0	0	1	0	1	1
105	cotton						1	0	0	1	0	1	1
105	cotton						1	0	0	1	0	1	1
105	cotton						1	0	0	1	0	1	1
105	cotton						1	0	0	1	0	1	1
105	cotton	85.2					1	0	0	1	0	1	1
105	cotton						1	0	0	1	0	1	1
105	cotton	63.3					1	0	0	1	0	1	1
105	cotton						1	0	0	1	0	1	1
105	cotton	56.3					1	0	0	1	0	1	1
105	cotton						1	0	0	1	0	1	1
105	cotton					105.8	1	0	0	1	0	1	1
105	cotton					213.2	1	0	0	1	0	1	1
105	cotton						1	0	0	1	0	1	1
105	cotton						1	0	0	1	0	1	1
105	cotton						1	0	0	1	0	1	1
105	cotton						1	0	0	1	0	1	1
105	cotton						1	0	0	1	0	1	1
105	cotton						1	0	0	1	0	1	1
105	cotton						1	0	0	1	0	1	1
105	cotton						1	0	0	1	0	1	1
105	cotton			-52.9			1	0	0	1	0	1	1
105	cotton						1	0	0	1	0	1	1
118	cotton		-25.0				1	0	0	0	0	1	1
118	cotton		-48.5				1	0	0	0	0	1	1
144	cotton	56.3					1	0	0	1	0	1	1
144	cotton						1	0	0	1	0	1	1
144	cotton				-17.3		1	0	0	1	0	1	1
144	cotton			-53.2			1	0	0	1	0	1	1
144	cotton						1	0	0	1	0	1	1
144	cotton						1	0	0	1	0	1	1
144	cotton						1	0	0	1	0	1	1

144	cotton	63.3				1	0	0	1	0	1	1
144	cotton					1	0	0	1	0	1	1
144	cotton					1	1	0	1	0	1	1
144	cotton				105.8	1	0	0	1	0	1	1
144	cotton	85.2				1	0	0	1	0	1	1
144	cotton					1	0	0	1	0	1	1
144	cotton					1	0	0	1	0	1	1
144	cotton			18.9		1	0	0	1	0	1	1
144	cotton			17.4		1	0	0	1	0	1	1
144	cotton					1	0	0	1	0	1	1
144	cotton		-52.9			1	0	0	1	0	1	1
144	cotton				213.2	1	0	0	1	0	1	1
144	cotton					1	0	0	1	0	1	1
144	cotton		-63.0			1	0	0	1	0	1	1
207	maize					1	0	0	0	0	1	1
207	maize					1	1	0	0	0	1	1
207	maize				21.8	1	0	0	0	0	1	1
207	maize					1	1	0	0	0	1	1
207	maize					1	1	0	0	0	1	1
207	maize			0.9		1	0	0	0	0	1	1
207	maize					1	0	0	0	0	1	1
207	maize					1	0	0	0	0	1	1
207	maize			-3.4		1	0	0	0	0	1	1
207	maize				-55.9	1	0	0	0	0	1	1
207	maize					1	0	0	0	0	1	1
207	maize		0.0			1	0	0	0	0	1	1
207	maize					1	0	0	0	0	1	1
207	maize	10.5				1	0	0	0	0	1	1
207	maize			0.8		1	0	0	0	0	1	1
207	maize					1	0	0	0	0	1	1
207	maize	25.0				1	0	0	0	0	1	1
207	maize					1	0	0	0	0	1	1
207	maize				83.3	1	0	0	0	0	1	1
207	maize					1	0	0	0	0	1	1
207	maize					1	0	0	0	0	1	1
207	maize					1	0	0	0	0	1	1
207	maize				71.9	1	0	0	0	0	1	1
207	maize					1	0	0	0	0	1	1
207	maize					1	1	0	0	0	1	1
207	maize			-0.7		1	0	0	0	0	1	1
207	maize		0.0			1	0	0	0	0	1	1
207	maize				154.1	1	0	0	0	0	1	1
207	maize				13.1	1	0	0	0	0	1	1
207	maize	11.1				1	0	0	0	0	1	1
207	maize					1	0	0	0	0	1	1
207	maize				-68.5	1	0	0	0	0	1	1
207	maize			-0.7		1	0	0	0	0	1	1
207	maize		0.0			1	0	0	0	0	1	1
207	maize				30.0	1	0	0	0	0	1	1
207	maize					1	0	0	0	0	1	1
207	maize	14.5				1	0	0	0	0	1	1
207	maize		0.0			1	0	0	0	0	1	1
207	maize	16.7				1	0	0	0	0	1	1
207	maize					1	0	0	0	0	1	1
207	maize				58.9	1	0	0	0	0	1	1
207	maize				45.2	1	0	0	0	0	1	1
207	maize					1	1	0	0	0	1	1
207	maize		0.0			1	0	0	0	0	1	1
207	maize					1	0	0	0	0	1	1
215	soybean	12.0				0	0	1	0	1	1	1
215	soybean	10.6				0	0	1	0	1	1	1
215	soybean	7.4				0	0	1	0	1	1	1
215	soybean	3.7				0	0	1	0	1	1	1
215	soybean	12.0				0	0	1	0	1	1	1
215	soybean	16.9				0	0	1	0	1	1	1
215	soybean	2.0				0	0	1	0	1	1	1
215	soybean	12.7				0	0	1	0	1	1	1

215	soybean	2.3					0	0	1	0	1	1	1
215	soybean	2.0					0	0	1	0	1	1	1
242	cotton	25.6					1	0	0	1	1	1	1
242	cotton	52.6					1	0	0	1	1	1	1
242	cotton	60.4					1	0	0	1	1	1	1
270	cotton		-66.1				1	0	0	1	0	1	1
312	soybean	0.6					0	0	0	0	1	1	1
334	cotton	21.8					1	0	0	0	1	1	1
334	cotton	2.5					1	0	0	0	1	1	1
334	cotton						1	0	0	0	1	1	1
334	cotton	-2.0					1	0	0	0	1	1	1
334	cotton						1	0	0	0	1	1	1
334	cotton						1	0	0	0	1	1	1
334	cotton	18.8					1	0	0	0	1	1	1
334	cotton						1	0	0	0	1	1	1
334	cotton	34.4					1	0	0	0	1	1	1
334	cotton						1	0	0	0	1	1	1
334	cotton						1	0	0	0	1	1	1
334	cotton						1	0	0	0	1	1	1
334	cotton						1	0	0	0	1	1	1
334	cotton	13.5					1	0	0	0	1	1	1
334	cotton	7.2					1	0	0	0	1	1	1
334	cotton	15.7					1	0	0	0	1	1	1
334	cotton	19.1					1	0	0	0	1	1	1
334	cotton						1	0	0	0	1	1	1
396	maize	1.1					1	0	0	0	1	1	1
396	maize	1.4					1	0	0	0	1	1	1
504	maize						1	1	0	0	0	1	1
504	maize				-1.3		1	0	0	0	0	1	1
504	maize						1	0	0	0	0	1	1
504	maize					14.4	1	0	0	0	0	1	1
504	maize						1	0	0	0	0	1	1
527	soybean	17.0					0	0	0	0	1	1	1
527	soybean	8.3					0	0	0	0	1	1	1
527	soybean	55.6					0	0	0	0	1	1	1
639	soybean				-5.2		0	0	0	0	0	0	0
639	soybean	28.8					0	0	0	0	0	0	0
639	soybean					-8.9	0	0	0	0	0	0	0
639	soybean	-4.3					0	0	0	0	0	0	0
643	soybean				12.1		0	0	1	0	1	1	1
643	soybean					-2.2	0	0	1	0	1	1	1
643	soybean	1.7					0	0	1	0	1	1	1
768	soybean	18.7					0	0	0	0	1	1	1
768	soybean			-34.2			0	0	0	0	1	1	1
768	soybean	-10.9					0	0	0	0	1	1	1
768	soybean			-27.9			0	0	0	0	1	1	1
768	soybean				-7.4		0	0	0	0	1	1	1
768	soybean	9.6					0	0	0	0	1	1	1
768	soybean						0	1	0	0	1	1	1
768	soybean	-20.0					0	0	0	0	1	1	1
768	soybean						0	1	0	0	1	1	1
768	soybean						0	1	0	0	1	1	1
768	soybean							1	0	0	1	1	1
768	soybean			-20.9			0	0	0	0	1	1	1
768	soybean						0	1	0	0	1	1	1
768	soybean	1.1					0	0	0	0	1	1	1
768	soybean	-6.6					0	0	0	0	1	1	1
768	soybean							1	0	0	1	1	1
768	soybean						0	1	0	0	1	1	1
768	soybean				-9.7		0	0	0	0	1	1	1
768	soybean						0	1	0	0	1	1	1
768	soybean							1	0	0	1	1	1
768	soybean							1	0	0	1	1	1
768	soybean			-14.3			0	0	0	0	1	1	1
768	soybean	-16.6					0	0	0	0	1	1	1
768	soybean				-2.8		0	0	0	0	1	1	1
768	soybean				-11.8		0	0	0	0	1	1	1

768	soybean						0	1	0	0	1	1	1
768	soybean	26.4					0	0	0	0	1	1	1
768	soybean						0	1	0	0	1	1	1
804	cotton			-82.2			1	0	0	1	0	1	1
804	cotton				-27.8		1	0	0	1	0	1	1
804	cotton	5.8					1	0	0	1	0	1	1
804	cotton		-80.6				1	0	0	1	0	1	1
856	soybean				-10.8		0	0	0	1	1	1	1
856	soybean			-86.8			0	0	0	1	1	1	1
856	soybean				-11.7		0	0	0	1	1	1	1
962	cotton						1	0	0	0	0	1	1
962	cotton	-4.0					0	0	0	0	0	1	1
962	cotton			0.6			1	0	0	0	0	1	1
962	soybean						0	0	0	0	0	1	1
962	soybean			-23.6			0	0	0	0	0	1	1
962	soybean	18.2					0	0	0	0	0	1	1
962	cotton			-12.8			0	0	0	0	0	1	1
962	cotton						0	0	0	0	0	1	1
962	cotton						1	0	0	0	0	1	1
962	cotton						0	0	0	0	0	1	1
962	soybean						0	0	0	0	0	1	1
962	cotton						0	0	0	0	0	1	1
962	cotton			-14.5			1	0	0	0	0	1	1
962	cotton	6.1					1	0	0	0	0	1	1
962	soybean						0	0	0	0	0	1	1
962	cotton						0	0	0	0	0	1	1
962	soybean	15.4					0	0	0	0	0	1	1
962	soybean	13.0					0	0	0	0	0	1	1
962	cotton	-7.9					1	0	0	0	0	1	1
962	soybean						0	0	0	0	0	1	1
962	cotton	-10.4					0	0	0	0	0	1	1
962	soybean			-27.8			0	0	0	0	0	1	1
962	cotton			-22.5			0	0	0	0	0	1	1
962	soybean			-33.0			0	0	0	0	0	1	1
962	soybean	-14.7					0	0	0	0	0	1	1
962	soybean			-41.4			0	0	0	0	0	1	1
1018	soybean							1	0	0	1	0	0
1018	soybean							1	0	0	1	0	0
1018	soybean	-5.3					0	0	0	0	1	0	0
1018	soybean	-5.9					0	0	0	0	1	0	0
1018	soybean	-2.8					0	0	0	0	1	0	0
1018	soybean							1	0	0	1	0	0
1018	soybean	-13.1					0	0	0	0	1	0	0
1018	soybean							1	0	0	1	0	0
1092	soybean						0	0	0	1	0	1	1
1092	soybean						0	1	0	1	0	1	1
1092	soybean						0	0	0	1	0	1	1
1092	soybean						0	1	0	1	0	1	1
1097	cotton		-9.1				1	0	0	1	0	1	1
1100	cotton					6.7	1	0	0	0	0	1	1
1100	cotton		-55.4				1	0	0	0	0	1	1
1100	cotton					74.2	1	0	0	0	0	1	1
1100	cotton		-76.2				1	0	0	0	0	1	1
1103	maize	38.2					0	0	0	1	0	1	1
1103	maize						1	0	0	1	0	1	1
1103	maize						1	0	0	1	0	1	1
1103	maize	1.8					1	0	0	1	0	1	1
1103	maize						0	0	0	1	0	1	1
1103	maize						0	0	0	1	0	1	1
1103	maize						0	0	0	1	0	1	1
1103	maize						1	0	0	1	0	1	1
1103	maize						0	0	0	1	0	1	1
1103	maize						0	0	0	1	0	1	1
1103	maize						0	0	0	1	0	1	1
1103	maize			-29.0			1	0	0	1	0	1	1
1103	maize						1	0	0	1	0	1	1
1103	maize						1	0	0	1	0	1	1

1103	maize						1	0	0	1	0	1	1
1103	maize					124.0	1	0	0	1	0	1	1
1103	maize						0	0	0	1	0	1	1
1103	maize						1	0	0	1	0	1	1
1103	maize					440.2	0	0	0	1	0	1	1
1103	maize						1	0	0	1	0	1	1
1103	maize	84.8					0	0	0	1	0	1	1
1103	maize						0	0	0	1	0	1	1
1103	maize						1	0	0	1	0	1	1
1103	maize	5.9					1	0	0	1	0	1	1
1109	cotton						1	0	0	1	0	1	1
1109	cotton						1	0	0	1	0	1	1
1109	cotton	47.6					1	0	0	1	0	1	1
1109	cotton	91.6					1	1	0	1	0	1	1
1109	cotton			-12.8			1	0	0	1	0	1	1
1109	cotton	27.0					1	1	0	1	0	1	1
1109	cotton						1	0	0	1	0	1	1
1109	cotton			-13.6			1	0	0	1	0	1	1
1109	cotton						1	0	0	1	0	1	1
1109	cotton						1	0	0	1	0	1	1
1109	cotton	47.1					1	0	0	1	0	1	1
1109	cotton						1	0	0	1	0	1	1
1109	cotton						1	0	0	1	0	1	1
1109	cotton						1	0	0	1	0	1	1
1115	cotton		-50.4				1	0	0	1	0	1	1
1115	cotton				16.8		1	0	0	1	0	1	1
1115	cotton				23.5		1	0	0	1	0	1	1
1115	cotton						1	0	0	1	0	1	1
1115	cotton						1	0	0	1	0	1	1
1115	cotton						1	0	0	1	0	1	1
1115	cotton						1	0	0	1	0	1	1
1115	cotton						1	0	0	1	0	1	1
1115	cotton		-51.1				1	0	0	1	0	1	1
1115	cotton	34.8					1	0	0	1	0	1	1
1115	cotton	34.2					1	0	0	1	0	1	1
1115	cotton						1	0	0	1	0	1	1
1115	cotton			-40.9			1	0	0	1	0	1	1
1115	cotton			-34.8			1	0	0	1	0	1	1
1115	cotton					128.7	1	0	0	1	0	1	1
1115	cotton			3.0			1	0	0	1	0	1	1
1115	cotton					70.3	1	0	0	1	0	1	1
1115	cotton		-21.3				1	0	0	1	0	1	1
1115	cotton						1	0	0	1	0	1	1
1115	cotton						1	0	0	1	0	1	1
1115	cotton				12.6		1	0	0	1	0	1	1
1115	cotton						1	0	0	1	0	1	1
1115	cotton						1	0	0	1	0	1	1
1115	cotton						1	0	0	1	0	1	1
1115	cotton						1	0	0	1	0	1	1
1115	cotton						1	0	0	1	0	1	1
1115	cotton						1	0	0	1	0	1	1
1115	cotton						1	0	0	1	0	1	1
1115	cotton						1	0	0	1	0	1	1
1115	cotton					62.6	1	0	0	1	0	1	1
1115	cotton						1	0	0	1	0	1	1
1115	cotton						1	0	0	1	0	1	1
1115	cotton	42.7					1	0	0	1	0	1	1
1130	soybean							1	1	0	0	1	1
1130	maize							1	1	0	0	1	1
1130	cotton							1	1	0	0	1	1
1141	cotton					49.2	1	0	0	1	0	1	1
1141	cotton						1	0	0	1	0	1	1
1141	cotton	61.6					1	1	0	1	0	1	1
1141	cotton						1	0	0	1	0	1	1
1141	cotton			-71.5			1	0	0	1	0	1	1
1141	cotton						1	0	0	1	0	1	1
1141	cotton					73.9	1	0	0	1	0	1	1

1141	cotton			2.1	1	0	0	1	0	1	1
1141	cotton				1	0	0	1	0	1	1
1141	cotton	39.1			1	1	0	1	0	1	1
1141	cotton	62.8			1	0	0	1	0	1	1
1141	cotton				1	0	0	1	0	1	1
1141	cotton				1	0	0	1	0	1	1
1141	cotton			14.7	1	0	0	1	0	1	1
1141	cotton	45.0			1	0	0	1	0	1	1
1141	cotton		-83.3		1	0	0	1	0	1	1
1147	maize				0	0	0	0	0	1	1
1147	soybean				0	0	0	0	0	1	1
1147	soybean				0	1	0	0	0	1	1
1147	cotton				0	1	0	0	0	1	1
1147	soybean				0	1	0	0	0	1	1
1147	cotton				0	0	0	0	0	1	1
1147	cotton				0	1	0	0	0	1	1
1159	maize	31.0			1	0	0	1	0	1	1
1159	maize	-3.0			0	0	0	1	0	1	1
1159	maize	21.0			0	0	0	1	0	1	1
1159	maize	32.0			1	0	0	1	0	1	1
1159	maize	9.3			0	0	0	1	0	1	1
1159	maize	14.0			1	0	0	1	0	1	1
1159	maize	4.0			1	0	0	1	0	1	1
1159	maize	32.0			0	0	0	1	0	1	1
1159	maize	94.0			0	0	0	1	0	1	1
1159	maize	11.9			0	0	0	1	0	1	1
1159	maize	10.0			0	0	0	1	0	1	1
1159	maize	25.0			1	0	0	1	0	1	1
1159	maize					1	0	1	0	1	1
1159	maize	29.0			1	0	0	1	0	1	1
1159	maize	4.2			1	0	0	1	0	1	1
1159	maize	10.3			0	0	0	1	0	1	1
1159	maize	94.0			0	0	0	1	0	1	1
1159	maize	10.1			0	0	0	1	0	1	1
1159	maize	22.0			1	0	0	1	0	1	1
1159	maize	21.0			1	0	0	1	0	1	1
1159	maize	8.0			0	0	0	1	0	1	1
1159	maize	21.0			1	0	0	1	0	1	1
1159	maize	12.0			0	0	0	1	0	1	1
1159	maize	9.0			0	0	0	1	0	1	1
1159	maize	10.0			0	0	0	1	0	1	1
1159	maize	16.0			1	0	0	1	0	1	1
1159	maize	8.0			1	0	0	1	0	1	1
1159	maize	33.0			0	0	0	1	0	1	1
1159	maize	22.0			1	0	0	1	0	1	1
1163	cotton	33.0			1	0	0	1	0	1	1
1163	cotton					1	0	1	0	1	1
1163	cotton					1	0	1	0	1	1
1163	cotton					1	0	1	0	1	1
1163	cotton		-77.0		1	0	0	1	0	1	1
1163	cotton					1	0	1	0	1	1
1163	cotton	34.0			1	0	0	1	0	1	1
1163	cotton		-33.0		1	0	0	1	0	1	1
1163	cotton	24.0			1	0	0	1	0	1	1
1163	cotton		-47.0		1	0	0	1	0	1	1
1163	cotton		-65.0		1	0	0	1	0	1	1
1163	cotton					1	0	1	0	1	1
1163	cotton					1	0	1	0	1	1
1163	cotton					1	0	1	0	1	1
1163	cotton					1	0	1	0	1	1
1163	cotton	9.0			1	0	0	1	0	1	1
1163	cotton	22.0			1	0	0	1	0	1	1
1163	cotton					1	0	1	0	1	1
1163	cotton					1	0	1	0	1	1
1163	cotton		-41.0		1	0	0	1	0	1	1
1164	cotton	49.5			1	0	0	1	0	1	1
1164	cotton				1	0	0	1	0	1	1

1164	cotton			95.0	1	0	0	1	0	1	1
1164	cotton		-48.8		1	0	0	1	0	1	1
1164	cotton				1	0	0	1	0	1	1
1164	cotton				1	0	0	1	0	1	1
1164	cotton				1	0	0	1	0	1	1
1164	cotton				1	0	0	1	0	1	1
1164	cotton				1	0	0	1	0	1	1
1164	cotton				1	0	0	1	0	1	1
1164	cotton		1.6		1	0	0	1	0	1	1
1164	cotton				1	0	0	1	0	1	1
1164	cotton	61.1			1	0	0	1	0	1	1
1164	cotton				1	0	0	1	0	1	1
1164	cotton			94.6	1	0	0	1	0	1	1
1164	cotton				1	0	0	1	0	1	1
1165	soybean					1	0	1	0	1	1
1165	soybean					1	0	1	0	1	1
1165	soybean					1	0	1	0	1	1
1165	maize					1	0	1	0	1	1
1165	soybean					1	0	1	0	1	1
1165	maize					1	0	1	0	1	1
1165	soybean					1	0	1	0	1	1
1165	maize					1	0	1	0	1	1
1165	maize					1	0	1	0	1	1
1165	soybean					1	0	1	0	1	1
1165	maize					1	0	1	0	1	1
1165	soybean					1	0	1	0	1	1
1165	maize					1	0	1	0	1	1
1165	maize					1	0	1	0	1	1
1165	maize					1	0	1	0	1	1
1165	maize					1	0	1	0	1	1
1165	soybean					1	0	1	0	1	1
1165	soybean					1	0	1	0	1	1
1165	soybean					1	0	1	0	1	1
1165	maize					1	0	1	0	1	1
1165	soybean					1	0	1	0	1	1
1165	soybean					1	0	1	0	1	1
1165	soybean					1	0	1	0	1	1
1165	soybean					1	0	1	0	1	1
1165	soybean					1	0	1	0	1	1
1165	maize					1	0	1	0	1	1
1165	soybean					1	0	1	0	1	1
1165	maize					1	0	1	0	1	1
1165	maize					1	0	1	0	1	1
1165	soybean					1	0	1	0	1	1
1165	maize					1	0	1	0	1	1
1165	maize					1	0	1	0	1	1
1165	soybean					1	0	1	0	1	1
1165	maize					1	0	1	0	1	1
1165	soybean					1	0	1	0	1	1
1165	soybean					1	0	1	0	1	1
1165	soybean					1	0	1	0	1	1
1165	maize					1	0	1	0	1	1
1165	maize					1	0	1	0	1	1
1165	maize					1	0	1	0	1	1
1165	maize					1	0	1	0	1	1
1165	soybean					1	0	1	0	1	1
1165	maize					1	0	1	0	1	1
1165	soybean					1	0	1	0	1	1
1165	maize					1	0	1	0	1	1
1165	maize					1	0	1	0	1	1
1165	soybean					1	0	1	0	1	1
1165	maize					1	0	1	0	1	1
1165	soybean					1	0	1	0	1	1
1165	soybean					1	0	1	0	1	1
1165	maize					1	0	1	0	1	1
1165	maize					1	0	1	0	1	1
1165	soybean					1	0	1	0	1	1

1165	soybean							1	0	1	0	1	1
1165	soybean							1	0	1	0	1	1
1165	maize							1	0	1	0	1	1
1165	maize							1	0	1	0	1	1
1165	maize							1	0	1	0	1	1
1165	maize							1	0	1	0	1	1
1165	soybean							1	0	1	0	1	1
1165	maize							1	0	1	0	1	1
1165	soybean							1	0	1	0	1	1
1165	soybean							1	0	1	0	1	1
1165	maize							1	0	1	0	1	1
1165	maize							1	0	1	0	1	1
1165	soybean							1	0	1	0	1	1
1165	maize							1	0	1	0	1	1
1165	maize							1	0	1	0	1	1
1165	soybean							1	0	1	0	1	1
1165	soybean							1	0	1	0	1	1
1165	maize							1	0	1	0	1	1
1165	soybean							1	0	1	0	1	1
1165	soybean							1	0	1	0	1	1
1165	maize							1	0	1	0	1	1
1165	maize							1	0	1	0	1	1
1165	soybean							1	0	1	0	1	1
1165	maize							1	0	1	0	1	1
1165	soybean							1	0	1	0	1	1
1165	soybean							1	0	1	0	1	1
1165	maize							1	0	1	0	1	1
1165	soybean							1	0	1	0	1	1
1165	soybean							1	0	1	0	1	1
1186	cotton	46.5					1	0	0	1	0	1	1
1186	cotton		-58.2				1	0	0	1	0	1	1
1188	cotton						1	0	0	1	0	1	1
1188	cotton		-15.0				1	0	0	1	0	1	1
1188	cotton					33.1	1	0	0	1	0	1	1
1188	cotton	24.3					1	0	0	1	0	1	1
1190	cotton						1	0	0	1	0	1	1
1190	cotton						1	0	0	1	0	1	1
1190	cotton			-90.7			1	0	0	1	0	1	1
1190	cotton						1	0	0	1	0	1	1
1190	cotton				0.7		1	0	0	1	0	1	1
1190	cotton						1	0	0	1	0	1	1
1190	cotton	18.2					1	0	0	1	0	1	1
1194	cotton						1	0	0	1	0	1	1
1194	cotton						1	0	0	1	0	1	1
1194	cotton						1	0	0	1	0	1	1
1194	cotton					258.1	1	0	0	1	0	1	1
1194	cotton	63.3					1	0	0	1	0	1	1
1194	cotton				7.8		1	0	0	1	0	1	1
1194	cotton						1	0	0	1	0	1	1
1194	cotton			-52.9			1	0	0	1	0	1	1
1194	cotton	56.3					1	0	0	1	0	1	1
1194	cotton				12.6		1	0	0	1	0	1	1
1194	cotton					105.8	1	0	0	1	0	1	1
1194	cotton			28.1			1	0	0	1	0	1	1
1194	cotton					213.2	1	0	0	1	0	1	1
1194	cotton						1	0	0	1	0	1	1
1194	cotton					194.2	1	0	0	1	0	1	1
1194	cotton	85.2					1	0	0	1	0	1	1
1194	cotton						1	0	0	1	0	1	1
1194	cotton						1	0	0	1	0	1	1
1194	cotton						1	0	0	1	0	1	1
1194	cotton						1	0	0	1	0	1	1
1194	cotton						1	0	0	1	0	1	1
1194	cotton						1	1	0	1	0	1	1
1194	cotton				-17.3		1	0	0	1	0	1	1
1194	cotton						1	0	0	1	0	1	1
1194	cotton						1	0	0	1	0	1	1

1194	cotton					1	0	0	1	0	1	1
1194	cotton					1	0	0	1	0	1	1
1194	cotton					1	0	0	1	0	1	1
1194	cotton					1	1	0	1	0	1	1
1194	cotton		-63.0			1	0	0	1	0	1	1
1194	cotton			-12.4		1	0	0	1	0	1	1
1194	cotton		-17.6			1	0	0	1	0	1	1
1194	cotton					1	0	0	1	0	1	1
1194	cotton			-4.3		1	0	0	1	0	1	1
1194	cotton					1	0	0	1	0	1	1
1194	cotton					1	0	0	1	0	1	1
1194	cotton	55.4				1	0	0	1	0	1	1
1194	cotton	100.5				1	0	0	1	0	1	1
1194	cotton		-77.8			1	0	0	1	0	1	1
1194	cotton					1	1	0	1	0	1	1
1194	cotton					1	0	0	1	0	1	1
1194	cotton		-53.2			1	0	0	1	0	1	1
1194	cotton					1	0	0	1	0	1	1
1194	cotton					1	0	0	1	0	1	1
1194	cotton			18.9		1	0	0	1	0	1	1
1194	cotton					1	0	0	1	0	1	1
1194	cotton			17.4		1	0	0	1	0	1	1
1195	cotton	20.0				1	0	0	0	1	1	1
1195	cotton		-66.7			1	0	0	0	1	1	1
1199	soybean	29.2				0	0	1	0	0	1	1
1199	soybean		-83.6			0	0	1	0	0	1	1
1199	soybean	33.3				0	0	1	0	0	1	1
1199	soybean			-18.7		0	0	1	0	0	1	1
1199	soybean				127.2	0	0	1	0	0	1	1
1199	soybean					0	0	1	0	0	1	1
1199	soybean	34.8				0	0	1	0	0	1	1
1199	soybean					0	0	1	0	0	1	1
1199	soybean					0	0	1	0	0	1	1
1199	soybean				183.9	0	0	1	0	0	1	1
1199	soybean					0	0	1	0	0	1	1
1199	soybean			-16.5		0	0	1	0	0	1	1
1199	soybean				185.4	0	0	1	0	0	1	1
1199	soybean					0	0	1	0	0	1	1
1199	soybean					0	0	1	0	0	1	1
1199	soybean			-43.1		0	0	1	0	0	1	1
1205	maize	54.4				1	0	0	1	0	1	1
1205	maize						1	0	1	0	1	1
1205	maize						1	0	1	0	1	1
1205	maize						1	0	1	0	1	1
1205	maize						1	0	1	0	1	1
1205	maize	54.9				1	0	0	1	0	1	1
1205	maize						1	0	1	0	1	1
1205	maize						1	0	1	0	1	1
1205	maize						1	0	1	0	1	1
1205	maize	96.8				1	0	0	1	0	1	1
1205	maize	62.0				1	0	0	1	0	1	1
1205	maize	21.0				1	0	0	1	0	1	1
1205	maize	30.0				1	0	0	1	0	1	1
1205	maize	62.0				1	0	0	1	0	1	1
1205	maize	34.0				1	0	0	1	0	1	1
1205	maize	52.3				1	0	0	1	0	1	1
1205	maize	32.0				1	0	0	1	0	1	1
1205	maize	26.0				1	0	0	1	0	1	1
1205	maize						1	0	1	0	1	1
1205	maize	85.4				1	0	0	1	0	1	1
1213	maize					0	1	1	0	0	1	1
1213	cotton					0	1	1	0	0	1	1
1213	soybean					0	1	1	0	0	1	1
1215	maize					1	0	0	1	0	1	1
1215	maize	25.5				1	0	0	1	0	1	1
1215	maize					1	0	0	1	0	1	1
1215	maize					1	0	0	1	0	1	1

1215	maize			-57.8			1	0	0	1	0	1	1
1215	maize						1	0	0	1	0	1	1
1215	maize		80.0				1	0	0	1	0	1	1
1215	maize						1	0	0	1	0	1	1
1215	maize		-80.0				1	0	0	1	0	1	1
1233	cotton	32.3					1	0	0	1	0	1	1
1233	cotton		-29.1				1	1	0	1	0	1	1
1233	cotton		-55.4				1	0	0	1	0	1	1
1233	cotton							1	0	1	0	1	1
1233	cotton	32.2					1	0	0	1	0	1	1
1233	cotton		-29.6				1	1	0	1	0	1	1
1233	cotton		-42.9				1	0	0	1	0	1	1
1239	cotton					76.8	1	0	0	1	0	1	1
1239	cotton						1	0	0	1	0	1	1
1239	cotton			-37.1			1	0	0	1	0	1	1
1239	cotton						1	0	0	1	0	1	1
1239	cotton						1	0	0	1	0	1	1
1239	cotton	17.5					1	0	0	1	0	1	1
1239	cotton	58.7					1	0	0	1	0	1	1
1239	cotton			-13.6			1	0	0	1	0	1	1
1239	cotton					10.9	1	0	0	1	0	1	1
1239	cotton						1	0	0	1	0	1	1
1241	cotton			-82.0			1	0	0	1	0	1	1
1241	cotton				-25.7		1	0	0	1	0	1	1
1241	cotton						1	1	0	1	0	1	1
1241	cotton		-80.0				1	0	0	1	0	1	1
1241	cotton						1	1	0	1	0	1	1
1241	cotton						1	1	0	1	0	1	1
1241	cotton						1	1	0	1	0	1	1
1241	cotton						1	0	0	1	0	1	1
1241	cotton		-66.7				1	0	0	1	0	1	1
1241	cotton		-80.6				1	0	0	1	0	1	1
1241	cotton						1	1	0	1	0	1	1
1241	cotton						1	1	0	1	0	1	1
1241	cotton						1	1	0	1	0	1	1
1241	cotton			-55.2			1	0	0	1	0	1	1
1241	cotton						1	0	0	1	0	1	1
1241	cotton						1	1	0	1	0	1	1
1241	cotton						1	1	0	1	0	1	1
1241	cotton						1	0	0	1	0	1	1
1241	cotton						1	1	0	1	0	1	1
1241	cotton						1	1	0	1	0	1	1
1241	cotton	3.3					1	0	0	1	0	1	1
1241	cotton						1	1	0	1	0	1	1
1243	cotton							1	0	1	0	1	1
1243	cotton						1	0	0	1	0	1	1
1243	cotton			-82.5			1	0	0	1	0	1	1
1243	cotton						1	0	0	1	0	1	1
1243	cotton		-57.7				1	0	0	1	0	1	1
1243	cotton						1	0	0	1	0	1	1
1243	cotton						1	0	0	1	0	1	1
1243	cotton	8.7					1	1	0	1	0	1	1
1243	cotton						1	0	0	1	0	1	1
1243	cotton		-65.9				1	1	0	1	0	1	1
1243	cotton	5.8					1	0	0	1	0	1	1
1243	cotton	54.7					1	0	0	1	0	1	1
1243	cotton						1	0	0	1	0	1	1
1243	cotton				-27.5		1	0	0	1	0	1	1
1243	cotton				-20.5		1	0	0	1	0	1	1
1243	cotton			-55.9			1	0	0	1	0	1	1
1243	cotton						1	0	0	1	0	1	1
1243	cotton		-80.6				1	0	0	1	0	1	1
1243	cotton						1	0	0	1	0	1	1
1243	cotton						1	0	0	1	0	1	1
1243	cotton						1	0	0	1	0	1	1
1243	cotton	10.9					1	0	0	1	0	1	1
1243	cotton						1	0	0	1	0	1	1

1243	cotton				1.3		1	0	0	1	0	1	1
1243	cotton			-58.1			1	0	0	1	0	1	1
1243	cotton						1	0	0	1	0	1	1
1243	cotton						1	0	0	1	0	1	1
1243	cotton						1	0	0	1	0	1	1
1243	cotton		-62.4				1	0	0	1	0	1	1
1243	cotton						1	0	0	1	0	1	1
1255	cotton			-47.6			1	0	0	1	0	1	1
1255	cotton			-57.1			1	0	0	1	0	1	1
1255	cotton						1	0	0	1	0	1	1
1255	cotton		-7.0				1	0	0	1	0	1	1
1255	cotton	45.3					1	0	0	1	0	1	1
1255	cotton	63.0					1	0	0	1	0	1	1
1255	cotton						1	0	0	1	0	1	1
1255	cotton						1	0	0	1	0	1	1
1255	cotton						1	0	0	1	0	1	1
1255	cotton		-32.3				1	0	0	1	0	1	1
1266	cotton	19.6					1	0	0	0	0	1	1
1266	cotton	-11.9					0	0	0	0	0	1	1
1266	cotton		5.0				1	0	0	0	0	1	1
1266	cotton		59.9				1	0	0	0	0	1	1
1266	cotton		-84.1				1	0	0	0	0	1	1
1266	cotton	8.6					1	0	0	0	0	1	1
1266	cotton		-22.5				0	0	0	0	0	1	1
1266	cotton		-23.4				0	0	0	0	0	1	1
1266	cotton		-57.5				1	0	0	0	0	1	1
1266	cotton	15.0					1	0	0	0	0	1	1
1266	cotton	26.1					1	0	0	0	0	1	1
1266	cotton		-22.9				1	0	0	0	0	1	1
1268	cotton				36.3		1	0	0	1	0	1	1
1268	cotton						1	0	0	1	0	1	1
1268	cotton						1	0	0	1	0	1	1
1268	cotton			-46.1			1	0	0	1	0	1	1
1268	cotton					53.8	1	0	0	1	0	1	1
1268	cotton	34.3					1	0	0	1	0	1	1
1268	cotton	32.4					1	0	0	1	0	1	1
1268	cotton				33.6		1	0	0	1	0	1	1
1268	cotton						1	0	0	1	0	1	1
1268	cotton					28.9	1	0	0	1	0	1	1
1278	soybean						0	1	0	0	0	1	1
1278	soybean						0	1	0	0	0	1	1
1291	soybean							1	0	0	0	1	1
1291	soybean						0	0	0	0	0	1	1
1291	soybean						0	0	0	0	0	1	1
1291	soybean							1	0	0	0	1	1
1363	soybean							1	0	0	0	1	1
1363	soybean							1	0	0	0	1	1
1363	soybean							1	0	0	0	1	1
1363	soybean					69.6	0	1	0	0	0	1	1
1363	soybean						0	1	0	0	0	1	1
1363	maize						1	1	0	0	0	1	1
1363	soybean							1	0	0	0	1	1
1363	soybean							1	0	0	0	1	1
1363	soybean							1	0	0	0	1	1
1363	soybean							1	0	0	0	1	1
1363	maize						1	1	0	0	0	1	1
1363	soybean							1	0	0	0	1	1
1363	soybean							1	0	0	0	1	1
1363	maize					-154.8	1	1	0	0	0	1	1
1363	soybean							1	0	0	0	1	1
1363	soybean					41.5	0	1	0	0	0	1	1
1363	maize						0	1	0	0	0	1	1
1363	soybean							1	0	0	0	1	1
1363	soybean							1	0	0	0	1	1
1363	soybean							1	0	0	0	1	1
1363	maize						0	1	0	0	0	1	1
1363	soybean						0	1	0	0	0	1	1

1363	soybean						1	0	0	0	1	1
1363	soybean						1	0	0	0	1	1
1363	soybean						1	0	0	0	1	1
1382	soybean				6.8	0	0	0	1	0	0	0
1382	soybean			-57.1		0	0	0	1	0	0	0
1382	soybean				4.5	0	0	0	1	0	0	0
1382	soybean				-1.8	0	0	0	1	0	0	0
1382	soybean					0	0	0	1	0	0	0
1382	soybean					0	0	0	1	0	0	0
1382	soybean					0	0	0	1	0	0	0
1382	soybean					0	0	0	1	0	0	0
1382	soybean				5.4	0	0	0	1	0	0	0
1382	soybean					0	0	0	1	0	0	0
1382	soybean			-55.9		0	0	0	1	0	0	0
1382	soybean			0.0		0	0	0	1	0	0	0
1382	soybean					0	0	0	1	0	0	0
1382	soybean	0.0				0	0	0	1	0	0	0
1382	soybean					0	0	0	1	0	0	0
1382	soybean			0.0		0	0	0	1	0	0	0
1382	soybean			-53.1		0	0	0	1	0	0	0
1382	soybean					0	0	0	1	0	0	0
1382	soybean				4.2	0	0	0	1	0	0	0
1382	soybean			-60.6		0	0	0	1	0	0	0
1382	soybean	0.0				0	0	0	1	0	0	0
1382	soybean			-57.3		0	0	0	1	0	0	0
1382	soybean			0.0		0	0	0	1	0	0	0
1382	soybean					0	0	0	1	0	0	0
1382	soybean			0.0		0	0	0	1	0	0	0
1382	soybean	2.9				0	0	0	1	0	0	0
1382	soybean					0	0	0	1	0	0	0
1382	soybean				6.3	0	0	0	1	0	0	0
1382	soybean	4.2				0	0	0	1	0	0	0
1382	soybean			0.0		0	0	0	1	0	0	0
1382	soybean	0.0				0	0	0	1	0	0	0
1382	soybean	0.0				0	0	0	1	0	0	0
1382	soybean	0.0				0	0	0	1	0	0	0
1382	soybean	0.0				0	0	0	1	0	0	0
1382	soybean			-57.5		0	0	0	1	0	0	0
1382	soybean			0.0		0	0	0	1	0	0	0
1382	soybean					0	0	0	1	0	0	0
1382	soybean					0	0	0	1	0	0	0
1458	cotton					0	1	0	0	1	1	1
1458	cotton					1	1	0	0	1	1	1
1550	cotton			-82.2		1	0	0	1	0	1	1
1550	cotton		-80.6			1	0	0	1	0	1	1
1550	cotton						1	0	1	0	1	1
1550	cotton	5.8				1	0	0	1	0	1	1
1550	cotton						1	0	1	0	1	1
1550	cotton		-58.2			1	1	0	1	0	1	1
1550	cotton	16.2				1	1	0	1	0	1	1
1550	cotton					1	0	0	1	0	1	1
1550	cotton					1	0	0	1	0	1	1
1559	maize						1	1	0	0	0	0
1559	maize						1	1	0	0	0	0
1559	maize						1	1	0	0	0	0
1559	maize						1	1	0	0	0	0
1559	maize						1	1	0	0	0	0
1559	maize						1	1	0	0	0	0
1559	maize						1	1	0	0	0	0
1559	maize						1	1	0	0	0	0
1559	maize						1	1	0	0	0	0
1559	maize						1	1	0	0	0	0
1570	cotton						1	0	1	0	0	0
1570	cotton					1	0	0	1	0	0	0
1570	cotton	5.8				1	0	0	1	0	0	0
1570	cotton		-58.2			1	1	0	1	0	0	0
1570	cotton		-80.6			1	0	0	1	0	0	0

1570	cotton						1	0	0	1	0	0	0
1570	cotton							1	0	1	0	0	0
1570	cotton			-82.2			1	0	0	1	0	0	0
1570	cotton	15.0					1	1	0	1	0	0	0
1593	cotton						1	0	0	1	0	1	1
1593	cotton	2.5					1	0	0	1	0	1	1
1593	cotton				-21.6		1	0	0	1	0	1	1
1593	cotton	4.3					1	0	0	1	0	1	1
1593	cotton			-80.3			1	0	0	1	0	1	1
1593	cotton						1	0	0	1	0	1	1
1618	soybean	16.1					0	0	0	0	0	1	1
1618	soybean			-33.9			0	0	0	0	0	1	1
1618	soybean			-4.5			0	0	0	0	0	1	1
1618	soybean	3.0					0	0	0	0	0	1	1
1618	soybean			-10.7			0	0	0	0	0	1	1
1618	soybean			8.8			0	0	0	0	0	1	1
1618	soybean		8.4				0	0	0	0	0	1	1
1618	soybean			-33.9			0	0	0	0	0	1	1
1618	soybean			-3.9			0	0	0	0	0	1	1
1618	soybean			-0.9			0	0	0	0	0	1	1
1618	soybean			-27.2			0	0	0	0	0	1	1
1618	soybean			-30.9			0	0	0	0	0	1	1
1618	soybean		-3.3				0	0	0	0	0	1	1
1618	soybean			-44.8			0	0	0	0	0	1	1
1618	soybean			-0.9			0	0	0	0	0	1	1
1618	soybean		-1.8				0	0	0	0	0	1	1
1618	soybean	14.3					0	0	0	0	0	1	1
1618	soybean		11.9				0	0	0	0	0	1	1
1618	soybean			8.9			0	0	0	0	0	1	1
1618	soybean		11.0				0	0	0	0	0	1	1
1618	soybean			-12.2			0	0	0	0	0	1	1
1631	cotton	-34.8					1	0	0	1	0	0	0
1631	cotton						1	0	0	1	0	0	0
1631	cotton			-6.6			1	0	0	1	0	0	0
1631	cotton						1	0	0	1	0	0	0
1631	cotton				11.4		1	0	0	1	0	0	0
1631	cotton			-7.1			1	0	0	1	0	0	0
1639	cotton	67.6					1	0	0	1	0	1	1
1639	cotton						1	0	0	1	0	1	1
1639	cotton						1	0	0	1	0	1	1
1639	cotton						1	0	0	1	0	1	1
1639	cotton	77.2					1	0	0	1	0	1	1
1639	cotton						1	0	0	1	0	1	1
1639	cotton						1	0	0	1	0	1	1
1639	cotton						1	0	0	1	0	1	1
1689	cotton	17.7					1	0	0	1	0	0	1
1689	cotton			-37.9			1	0	0	1	0	0	1
1689	cotton	59.8					1	0	0	1	0	0	1
1689	cotton						1	0	0	1	0	0	1
1689	cotton			-12.5			1	0	0	1	0	0	1
1689	cotton					10.9	1	0	0	1	0	0	1
1697	cotton						1	0	0	1	0	1	1
1697	cotton						1	0	0	1	0	1	1
1697	cotton			-53.2			1	0	0	1	0	1	1
1697	cotton	85.2					1	0	0	1	0	1	1
1697	cotton						1	0	0	1	0	1	1
1697	cotton	63.3					1	0	0	1	0	1	1
1697	cotton						1	0	0	1	0	1	1
1697	cotton	56.3					1	0	0	1	0	1	1
1697	cotton						1	0	0	1	0	1	1
1697	cotton			-63.0			1	0	0	1	0	1	1
1697	cotton						1	0	0	1	0	1	1
1697	cotton						1	0	0	1	0	1	1
1697	cotton					105.8	1	0	0	1	0	1	1
1697	cotton						1	0	0	1	0	1	1
1697	cotton						1	0	0	1	0	1	1
1697	cotton						1	0	0	1	0	1	1

1697	cotton						1	0	0	1	0	1	1
1697	cotton			-52.9			1	0	0	1	0	1	1
1697	cotton					213.2	1	0	0	1	0	1	1
1697	cotton						1	0	0	1	0	1	1
1734	cotton	61.1					1	0	0	1	0	1	1
1734	cotton							1	0	1	0	1	1
1734	cotton		-62.3				1	0	0	1	0	1	1
1778	cotton						1	1	0	1	0	1	1
1797	cotton					155.3	1	0	0	1	1	1	1
1797	cotton	2.3					1	0	0	1	1	1	1
1797	cotton						1	0	0	1	1	1	1
1797	cotton						1	0	0	1	1	1	1
1797	cotton						1	0	0	1	1	1	1
1797	cotton	2.6					1	0	0	1	1	1	1
1797	cotton				-27.2		1	0	0	1	1	1	1
1797	cotton				-22.8		1	0	0	1	1	1	1
1797	cotton				-21.6		1	0	0	1	1	1	1
1797	cotton	0.5					1	0	0	1	1	1	1
1809	cotton	3.9					1	0	0	1	0	1	1
1809	cotton					-1.3	1	0	0	1	0	1	1
1809	cotton	39.8					1	0	0	1	0	1	1
1809	cotton						1	0	0	1	0	1	1
1809	cotton						1	0	0	1	0	1	1
1809	cotton						1	0	0	1	0	1	1
1809	cotton						1	0	0	1	0	1	1
1809	cotton						1	0	0	1	0	1	1
1809	cotton						1	0	0	1	0	1	1
1809	cotton					57.7	1	0	0	1	0	1	1
1813	cotton							1	0	1	0	1	1
1813	cotton							1	0	1	0	1	1
1813	cotton			-56.4			1	0	0	1	0	1	1
1813	cotton							1	0	1	0	1	1
1813	cotton	18.5					1	0	0	1	0	1	1
1813	cotton							1	0	1	0	1	1
1813	cotton							1	0	1	0	1	1
1813	cotton	13.8					1	0	0	1	0	1	1
1813	cotton							1	0	1	0	1	1
1813	cotton			-59.2			1	0	0	1	0	1	1
1813	cotton	45.8					1	0	0	1	0	1	1
1813	cotton							1	0	1	0	1	1
1813	cotton							1	0	1	0	1	1
1813	cotton							1	0	1	0	1	1
1813	cotton			-24.9			1	0	0	1	0	1	1
1815	cotton						1	0	0	1	0	1	1
1815	cotton			-39.9			1	0	0	1	0	1	1
1815	cotton				19.6		1	0	0	1	0	1	1
1815	cotton						1	0	0	1	0	1	1
1815	cotton						1	0	0	1	0	1	1
1815	cotton						1	0	0	1	0	1	1
1815	cotton							1	0	1	0	1	1
1815	cotton			-83.0			1	0	0	1	0	1	1
1815	cotton						1	0	0	1	0	1	1
1815	cotton					452.6	1	0	0	1	0	1	1
1815	cotton		-61.1				1	1	0	1	0	1	1
1815	cotton						1	0	0	1	0	1	1
1815	cotton	83.7					1	0	0	1	0	1	1
1842	cotton						1	0	0	1	0	0	0
1842	cotton				17.3		1	0	0	1	0	0	0
1842	cotton			-12.3			1	0	0	1	0	0	0
1842	cotton						1	0	0	1	0	0	0
1842	cotton						1	0	0	1	0	0	0
1842	cotton	3.4					1	0	0	1	0	0	0
1842	cotton				8.1		1	0	0	1	0	0	0
1842	cotton	5.4					1	0	0	1	0	0	0
1842	cotton					-8.9	1	0	0	1	0	0	0
1842	cotton						1	0	0	1	0	0	0
1864	cotton	9.8					1	0	0	0	0	0	1

1864	cotton		-7.7				1	0	0	0	0	0	1
1864	cotton						1	1	0	0	0	0	1
1864	cotton						1	1	0	0	0	0	1
1872	soybean						0	0	0	1	0	1	1
1872	soybean			-43.2			0	0	0	1	0	1	1
1872	soybean				-9.7		0	0	0	1	0	1	1
1872	soybean					8.5	0	0	0	1	0	1	1
1872	soybean		16.8				0	0	0	1	0	1	1
1872	soybean	0.3					0	0	0	1	0	1	1
1880	cotton							1	0	1	0	1	1
1880	cotton							1	0	1	0	1	1
1880	cotton							1	0	1	0	1	1
1903	cotton						1	0	0	1	0	1	1
1903	cotton	74.9					1	0	0	1	0	1	1
1903	cotton						1	0	0	1	0	1	1
1903	cotton						1	0	0	1	0	1	1
1903	cotton						1	0	0	1	0	1	1
1903	cotton					68.3	1	0	0	1	0	1	1
1903	cotton						1	0	0	1	0	1	1
1903	cotton						1	0	0	1	0	1	1
1903	cotton						1	0	0	1	0	1	1
1903	cotton						1	0	0	1	0	1	1
1903	cotton			-92.2			1	0	0	1	0	1	1
1903	cotton			-58.6			1	0	0	1	0	1	1
1903	cotton						1	0	0	1	0	1	1
1903	cotton	18.0					1	0	0	1	0	1	1
1903	cotton						1	0	0	1	0	1	1
1903	cotton					91.8	1	0	0	1	0	1	1
1903	cotton					73.0	1	0	0	1	0	1	1
1903	cotton	39.1					1	0	0	1	0	1	1
1903	cotton					100.9	1	0	0	1	0	1	1
1903	cotton	84.0					1	0	0	1	0	1	1
1903	cotton						1	0	0	1	0	1	1
1903	cotton						1	0	0	1	0	1	1
1903	cotton						1	0	0	1	0	1	1
1903	cotton			-83.3			1	0	0	1	0	1	1
1903	cotton			-77.4			1	0	0	1	0	1	1
1903	cotton	40.9					1	0	0	1	0	1	1
1903	cotton						1	0	0	1	0	1	1
1903	cotton						1	0	0	1	0	1	1
1903	cotton						1	0	0	1	0	1	1
1903	cotton					45.5	1	0	0	1	0	1	1
1903	cotton						1	0	0	1	0	1	1
1903	cotton						1	0	0	1	0	1	1
1903	cotton					43.4	1	0	0	1	0	1	1
1903	cotton	63.0					1	0	0	1	0	1	1
1903	cotton						1	0	0	1	0	1	1
1903	cotton			-83.4			1	0	0	1	0	1	1
1903	cotton						1	0	0	1	0	1	1
1903	cotton			-63.8			1	0	0	1	0	1	1
1903	cotton						1	0	0	1	0	1	1
1903	cotton			-86.7			1	0	0	1	0	1	1
1903	cotton						1	0	0	1	0	1	1
1903	cotton					37.1	1	0	0	1	0	1	1
1903	cotton						1	0	0	1	0	1	1
1903	cotton						1	0	0	1	0	1	1
1903	cotton	34.9					1	0	0	1	0	1	1
1903	cotton			-71.6			1	0	0	1	0	1	1
1903	cotton					14.4	1	0	0	1	0	1	1
1903	cotton	60.0					1	0	0	1	0	1	1
1908	cotton						1	0	0	1	0	1	1
1908	cotton				6.1		1	0	0	1	0	1	1
1908	cotton			-38.1			1	0	0	1	0	1	1
1908	cotton	17.6					1	0	0	1	0	1	1
1908	cotton						1	0	0	1	0	1	1
1908	cotton					65.4	1	0	0	1	0	1	1
1919	maize	-2.7					1	0	0	0	1	1	1

1919	maize	0.7					1	0	0	0	1	1	1
1919	maize	0.5					1	0	0	0	1	1	1
1919	maize	-3.0					1	0	0	0	1	1	1
1919	maize	-12.8					1	0	0	0	1	1	1
1919	maize	1.0					1	0	0	0	1	1	1
1919	maize	-10.5					1	0	0	0	1	1	1
1919	maize	0.0					1	0	0	0	1	1	1
1919	maize	-7.7					1	0	0	0	1	1	1
1919	maize	4.2					1	0	0	0	1	1	1
1919	maize	-3.7					1	0	0	0	1	1	1
1919	maize	-14.8					1	0	0	0	1	1	1
1919	maize	15.4					1	0	0	0	1	1	1
1919	maize	0.0					1	0	0	0	1	1	1
1919	maize	1.5					1	0	0	0	1	1	1
1919	maize	-7.1					1	0	0	0	1	1	1
1922	cotton						1	0	0	1	0	0	0
1922	cotton						1	0	0	1	0	0	0
1922	cotton					21.9	1	0	0	1	0	0	0
1922	cotton						1	0	0	1	0	0	0
1922	cotton						1	0	0	1	0	0	0
1922	cotton						1	0	0	1	0	0	0
1922	cotton				28.4		1	0	0	1	0	0	0
1922	cotton						1	0	0	1	0	0	0
1922	cotton					-20.3	1	0	0	1	0	0	0
1922	cotton						1	0	0	1	0	0	0
1922	cotton	14.9					1	0	0	1	0	0	0
1922	cotton						1	0	0	1	0	0	0
1922	cotton						1	0	0	1	0	0	0
1922	cotton						1	1	0	1	0	0	0
1922	cotton			-22.5			1	0	0	1	0	0	0
1922	cotton						1	0	0	1	0	0	0
1922	cotton					-31.4	1	0	0	1	0	0	0
1922	cotton						1	0	0	1	0	0	0
1922	cotton			-61.2			1	0	0	1	0	0	0
1922	cotton	8.1					1	0	0	1	0	0	0
1922	cotton						1	0	0	1	0	0	0
1922	cotton	2.0					1	0	0	1	0	0	0
1922	cotton				16.9		1	0	0	1	0	0	0
1922	cotton				8.4		1	0	0	1	0	0	0
1922	cotton						1	0	0	1	0	0	0
1922	cotton	14.6					1	0	0	1	0	0	0
1922	cotton					32.2	1	0	0	1	0	0	0
1922	cotton			-56.0			1	0	0	1	0	0	0
1922	cotton						1	0	0	1	0	0	0
1922	cotton						1	0	0	1	0	0	0
1926	cotton	65.4					1	0	0	1	0	1	1
1926	cotton		-2.1				1	0	0	1	0	1	1
1926	cotton	57.4					1	0	0	1	0	1	1
1926	cotton		-23.9				1	0	0	1	0	1	1
1929	soybean							1	0	0	0	0	0
1929	soybean							1	0	0	0	0	0
1929	maize			-24.8			0	0	0	0	0	0	0
1929	soybean							1	0	0	0	0	0
1929	soybean							1	0	0	0	0	0
1945	maize							1	1	1	0	1	1
1945	maize			-64.5			1	0	1	1	0	1	1
1945	maize							1	1	1	0	1	1
1945	maize							1	1	1	0	1	1
1945	maize							1	1	1	0	1	1
1945	maize							1	1	1	0	1	1
1945	maize			-59.1			1	0	1	1	0	1	1
1945	maize							1	1	1	0	1	1
1945	maize							1	1	1	0	1	1
1945	maize	10.6					1	0	1	1	0	1	1
1945	maize							1	1	1	0	1	1
1945	maize							1	1	1	0	1	1
1945	maize							1	1	1	0	1	1

1945	maize							1	1	1	0	1	1
1945	maize			-50.6			1	0	1	1	0	1	1
1945	maize	11.0					1	0	1	1	0	1	1
1945	maize	11.0					1	0	1	1	0	1	1
1945	maize	10.6					1	0	1	1	0	1	1
1945	maize							1	1	1	0	1	1
1945	maize							1	1	1	0	1	1
1945	maize							1	1	1	0	1	1
1945	maize							1	1	1	0	1	1
1945	maize							1	1	1	0	1	1
1945	maize			-80.4			1	0	1	1	0	1	1
1966	cotton	35.5					1	0	0	1	0	1	1
1966	cotton	28.8					1	0	0	1	0	1	1
1966	cotton	80.7					1	0	0	1	0	1	1
1970	maize	-2.1					1	0	0	0	1	1	1
1970	maize	13.4					1	0	0	0	1	1	1
1970	maize	10.3					1	0	0	0	1	1	1
1970	maize	38.6					1	0	0	0	1	1	1
1970	maize	-12.9					1	0	0	0	1	1	1
1970	maize	10.7					1	0	0	0	1	1	1
1970	maize	22.6					1	0	0	0	1	1	1
1970	maize	-12.2					1	0	0	0	1	1	1
1970	maize	20.9					1	0	0	0	1	1	1
1970	maize	13.8					1	0	0	0	1	1	1
1980	soybean						0	1	0	0	0	0	1
1995	maize							1	0	0	0	0	0
1995	soybean			-43.7			0	0	0	0	0	0	0
1995	soybean		-34.3				0	0	0	0	0	0	0
1995	soybean							1	0	0	0	0	0
1995	soybean							1	0	0	0	0	0
1999	maize						1	0	0	1	0	1	1
1999	maize	38.2					1	0	0	1	0	1	1
1999	maize	-1.4					1	0	0	1	0	1	1
1999	maize						1	0	0	1	0	1	1
1999	maize	1.5					1	0	0	1	0	1	1
2002	cotton						1	0	0	1	0	1	1
2002	cotton					79.1	1	0	0	1	0	1	1
2002	cotton		-2.3				1	0	0	1	0	1	1
2002	cotton						1	0	0	1	0	1	1
2002	cotton						1	0	0	1	0	1	1
2002	cotton						1	0	0	1	0	1	1
2002	cotton						1	0	0	1	0	1	1
2002	cotton						1	0	0	1	0	1	1
2002	cotton							1	0	1	0	1	1
2002	cotton				49.3		1	0	0	1	0	1	1
2002	cotton						1	0	0	1	0	1	1
2002	cotton						1	0	0	1	0	1	1
2002	cotton			4.7			1	0	0	1	0	1	1
2002	cotton					77.7	1	0	0	1	0	1	1
2002	cotton				34.8		1	0	0	1	0	1	1
2002	cotton				29.2		1	0	0	1	0	1	1
2002	cotton						1	0	0	1	0	1	1
2002	cotton						1	0	0	1	0	1	1
2002	cotton						1	0	0	1	0	1	1
2002	cotton		5.6				1	0	0	1	0	1	1
2002	cotton						1	0	0	1	0	1	1
2002	cotton			8.4			1	0	0	1	0	1	1
2002	cotton						1	0	0	1	0	1	1
2002	cotton			16.8			1	0	0	1	0	1	1
2002	cotton						1	0	0	1	0	1	1
2002	cotton		-5.4				1	0	0	1	0	1	1
2002	cotton						1	0	0	1	0	1	1
2002	cotton					91.3	1	0	0	1	0	1	1
2002	cotton						1	0	0	1	0	1	1
2002	cotton						1	0	0	1	0	1	1
2002	cotton						1	0	0	1	0	1	1
2002	cotton						1	0	0	1	0	1	1

2002	cotton						1	0	0	1	0	1	1
2002	cotton						1	0	0	1	0	1	1
2002	cotton						1	0	0	1	0	1	1
2002	cotton						1	0	0	1	0	1	1
2002	cotton						1	0	0	1	0	1	1
2002	cotton						1	0	0	1	0	1	1
2002	cotton						1	0	0	1	0	1	1
2002	cotton						1	0	0	1	0	1	1
2007	cotton							1	1	1	0	1	1
2007	cotton		-26.7				1	0	1	1	0	1	1
2007	cotton							1	1	1	0	1	1
2007	cotton						1	0	1	1	0	1	1
2007	cotton			13.2			1	0	1	1	0	1	1
2007	cotton						1	0	1	1	0	1	1
2007	cotton							1	1	1	0	1	1
2007	cotton		16.6				1	0	1	1	0	1	1
2007	cotton			-27.0			1	0	1	1	0	1	1
2007	cotton							1	1	1	0	1	1
2025	cotton	10.4					1	0	0	1	0	1	1
2025	cotton	-1.1					1	0	0	1	0	1	1
2025	cotton	27.5					1	0	0	1	0	1	1
2025	cotton	-9.7					1	0	0	1	0	1	1
2025	cotton	16.5					1	0	0	1	0	1	1
2029	cotton	58.9					1	1	0	1	0	1	1
2029	cotton	31.9					1	0	0	1	0	1	1
2029	cotton	46.8					1	1	0	1	0	1	1
2029	cotton		-75.6				1	0	0	1	0	1	1
2029	cotton		-21.2				1	0	0	1	0	1	1
2029	cotton				5.4		1	0	0	1	0	1	1
2029	cotton						1	0	0	1	0	1	1
2029	cotton			-18.9			1	0	0	1	0	1	1
2029	cotton					56.0	1	0	0	1	0	1	1
2029	cotton						1	0	0	1	0	1	1
2029	cotton				18.5		1	0	0	1	0	1	1
2029	cotton	34.2					1	0	0	1	0	1	1
2029	cotton	72.9					1	0	0	1	0	1	1
2029	cotton					-40.1	1	0	0	1	0	1	1
2029	cotton						1	0	0	1	0	1	1
2029	cotton				13.1		1	0	0	1	0	1	1
2029	cotton		-32.2				1	0	0	1	0	1	1
2029	cotton	26.7					1	1	0	1	0	1	1
2029	cotton				15.4		1	0	0	1	0	1	1
2029	cotton			-48.9			1	0	0	1	0	1	1
2029	cotton						1	0	0	1	0	1	1
2029	cotton					228.7	1	0	0	1	0	1	1
2029	cotton		-50.4				1	0	0	1	0	1	1
2029	cotton			-43.8			1	0	0	1	0	1	1
2029	cotton	43.0					1	0	0	1	0	1	1
2029	cotton		-38.4				1	0	0	1	0	1	1
2029	cotton	-3.1					1	0	0	1	0	1	1
2029	cotton			-40.9			1	0	0	1	0	1	1
2029	cotton					69.0	1	0	0	1	0	1	1
2029	cotton						1	0	0	1	0	1	1
2029	cotton					172.2	1	0	0	1	0	1	1
2029	cotton						1	0	0	1	0	1	1
2029	cotton						1	0	0	1	0	1	1
2029	cotton		-43.9				1	0	0	1	0	1	1
2029	cotton				16.8		1	0	0	1	0	1	1
2029	cotton						1	0	0	1	0	1	1
2029	cotton			-72.9			1	0	0	1	0	1	1
2032	cotton						1	0	0	1	0	0	1
2032	cotton			0.0			1	0	0	1	0	0	1
2068	cotton	42.6					1	1	0	1	0	0	0
2068	cotton						1	0	0	1	0	0	0
2068	cotton						1	0	0	1	0	0	0
2068	cotton						1	0	0	1	0	0	0
2068	cotton						1	0	0	1	0	0	0

2068	cotton	2.3					1	0	0	1	0	0	0
2068	cotton						1	0	0	1	0	0	0
2068	cotton						1	0	0	1	0	0	0
2075	cotton					95.0	1	0	0	1	0	1	1
2075	cotton					94.6	1	0	0	1	0	1	1
2075	cotton				34.7		1	0	0	1	0	1	1
2075	cotton	48.1					1	0	0	1	0	1	1
2075	cotton						1	0	0	1	0	1	1
2075	cotton				4.7		1	0	0	1	0	1	1
2075	cotton	65.2					1	0	0	1	0	1	1
2075	cotton						1	0	0	1	0	1	1
2101	cotton						1	0	0	1	0	1	1
2101	cotton			-42.4			1	0	0	1	0	1	1
2101	cotton						1	0	0	1	0	1	1
2101	cotton	3.1					1	0	0	1	0	1	1
2101	cotton						1	0	0	1	0	1	1
2101	cotton						1	0	0	1	0	1	1
2101	cotton		-68.5				1	0	0	1	0	1	1
2101	cotton			-62.1			1	0	0	1	0	1	1
2101	cotton	15.4					1	0	0	1	0	1	1
2101	cotton						1	1	0	1	0	1	1
2101	cotton						1	0	0	1	0	1	1
2101	cotton	10.2					1	0	0	1	0	1	1
2101	cotton			-45.4			1	0	0	1	0	1	1
2101	cotton						1	1	0	1	0	1	1
2101	cotton							1	0	1	0	1	1
2101	cotton		-54.9				1	0	0	1	0	1	1
2101	cotton						1	0	0	1	0	1	1
2101	cotton		-61.1				1	0	0	1	0	1	1
2101	cotton	26.4					1	0	0	1	0	1	1
2101	cotton						1	0	0	1	0	1	1
2101	cotton				0.3		1	0	0	1	0	1	1
2101	cotton				-2.4		1	0	0	1	0	1	1
2101	cotton						1	0	0	1	0	1	1
2101	cotton						1	0	0	1	0	1	1
2101	cotton				-1.0		1	0	0	1	0	1	1
2101	cotton						1	0	0	1	0	1	1
2101	cotton		-49.9				1	0	0	1	0	1	1
2101	cotton				-2.4		1	0	0	1	0	1	1
2111	cotton						0	0	0	0	0	0	1
2111	soybean						0	0	0	0	0	0	1
2111	soybean						0	1	0	0	0	0	1
2111	maize						0	0	0	0	0	0	1
2136	cotton							1	0	1	0	1	1
2136	cotton							1	0	1	0	1	1
2136	cotton							1	0	1	0	1	1
2136	cotton							1	0	1	0	1	1
2147	cotton	46.2					1	0	0	1	0	1	1
2147	cotton						1	0	0	1	0	1	1
2147	cotton		-33.3				1	0	0	1	0	1	1
2147	cotton						1	0	0	1	0	1	1
2181	cotton					86.9	1	0	0	1	0	1	1
2181	cotton				-1.0		1	0	0	1	0	1	1
2181	cotton						1	0	0	1	0	1	1
2181	cotton	53.6					1	0	0	1	0	1	1
2181	cotton						1	0	0	1	0	1	1
2181	cotton				-2.2		1	0	0	1	0	1	1
2181	cotton	54.3					1	0	0	1	0	1	1
2181	cotton					90.0	1	0	0	1	0	1	1
2187	cotton							1	0	0	0	1	1
2187	cotton							1	0	0	0	1	1
2187	cotton							1	0	0	0	1	1
2194	cotton						1	0	0	0	0	1	1
2194	cotton						1	0	0	0	0	1	1
2194	cotton						1	0	0	0	0	1	1
2194	cotton						1	0	0	0	0	1	1
2196	maize							1	0	0	0	0	0

2196	maize					1	0	0	0	0	0
2196	maize					1	0	0	0	0	0
2196	maize	-1.3			1	0	0	0	0	0	0
2196	maize					1	0	0	0	0	0
2196	maize				1	0	0	0	0	0	0
2196	maize					1	0	0	0	0	0
2196	maize					1	0	0	0	0	0
2196	maize					1	0	0	0	0	0
2196	maize				1	0	0	0	0	0	0
2196	maize					1	0	0	0	0	0
2196	maize					1	0	0	0	0	0
2196	maize					1	0	0	0	0	0
2196	maize					1	0	0	0	0	0
2196	maize					1	0	0	0	0	0
2196	maize					1	0	0	0	0	0
2196	maize					1	0	0	0	0	0
2196	maize					1	0	0	0	0	0
2196	maize					1	0	0	0	0	0
2196	maize				1	0	0	0	0	0	0
2196	maize	0.5			1	0	0	0	0	0	0
2196	maize	3.7			1	0	0	0	0	0	0
2196	maize					1	0	0	0	0	0
2196	maize	10.9			1	0	0	0	0	0	0
2196	maize				1	0	0	0	0	0	0
2196	maize					1	0	0	0	0	0
2196	maize				1	0	0	0	0	0	0
2196	maize				1	0	0	0	0	0	0
2196	maize		-62.5		1	0	0	0	0	0	0
2196	maize				1	0	0	0	0	0	0
2196	maize					1	0	0	0	0	0
2196	maize				1	0	0	0	0	0	0
2196	maize				1	0	0	0	0	0	0
2196	maize					1	0	0	0	0	0
2196	maize					1	0	0	0	0	0
2196	maize					1	0	0	0	0	0
2196	maize					1	0	0	0	0	0
2196	maize					1	0	0	0	0	0
2196	maize					1	0	0	0	0	0
2196	maize				1	0	0	0	0	0	0
2196	maize		-71.6		1	0	0	0	0	0	0
2196	maize					1	0	0	0	0	0
2196	maize					1	0	0	0	0	0
2196	maize	11.6			1	0	0	0	0	0	0
2196	maize					1	0	0	0	0	0
2196	maize				1	0	0	0	0	0	0
2196	maize	1.8			1	0	0	0	0	0	0
2196	maize					1	0	0	0	0	0
2196	maize	12.1			1	0	0	0	0	0	0
2196	maize	4.3			1	0	0	0	0	0	0
2196	maize				1	0	0	0	0	0	0
2196	maize					1	0	0	0	0	0
2196	maize	10.0			1	0	0	0	0	0	0
2196	maize					1	0	0	0	0	0
2196	maize				1	0	0	0	0	0	0
2196	maize					1	0	0	0	0	0
2196	maize					1	0	0	0	0	0
2196	maize		-70.3		1	0	0	0	0	0	0
2196	maize				1	0	0	0	0	0	0
2196	maize					1	0	0	0	0	0
2196	maize					1	0	0	0	0	0
2196	maize	11.6			1	0	0	0	0	0	0
2196	maize					1	0	0	0	0	0
2196	maize				1	0	0	0	0	0	0
2199	cotton			-12.8	1	0	0	1	0	1	1
2199	cotton				1	0	0	1	0	1	1
2199	cotton				1	0	0	1	0	1	1
2199	cotton		-72.0		1	0	0	1	0	1	1

2199	cotton	22.3					1	0	0	1	0	1	1
2210	cotton				-4.7		1	0	0	1	0	0	0
2210	cotton			-80.0			1	0	0	1	0	0	0
2210	cotton	-45.4					1	0	0	1	0	0	0
2210	cotton			-45.4			1	0	0	1	0	0	0
2210	cotton			-43.1			1	0	0	1	0	0	0
2210	cotton					-90.4	1	0	0	1	0	0	0
2210	cotton				-12.9		1	0	0	1	0	0	0
2210	cotton	-43.1					1	0	0	1	0	0	0
2219	cotton		-59.5				1	0	0	1	0	1	1
2219	cotton		-51.1				1	0	0	1	0	1	1
2219	cotton		-46.0				1	0	0	1	0	1	1
2219	cotton	32.0					1	0	0	1	0	1	1
2219	cotton	34.2					1	0	0	1	0	1	1
2219	cotton		-50.4				1	0	0	1	0	1	1
2219	cotton	35.0					1	0	0	1	0	1	1
2219	cotton	39.1					1	0	0	1	0	1	1
2232	cotton		-67.1				1	0	0	1	0	1	1
2232	cotton	22.0					1	0	0	1	0	1	1
2232	cotton							1	0	1	0	1	1
2278	cotton					128.7	1	0	0	1	0	0	1
2278	cotton					69.0	1	0	0	1	0	0	1
2278	cotton		-21.3				1	0	0	1	0	0	1
2278	cotton	35.0					1	0	0	1	0	0	1
2278	cotton	34.2					1	0	0	1	0	0	1
2278	cotton		-51.1				1	0	0	1	0	0	1
2278	cotton	42.7					1	0	0	1	0	0	1
2278	cotton		-50.4				1	0	0	1	0	0	1
2278	cotton					70.3	1	0	0	1	0	0	1
2326	maize	36.4					1	1	0	0	0	0	1
2326	maize	44.5					1	1	0	0	0	0	1
2332	cotton	43.7					1	0	0	1	1	1	1
2332	cotton	45.4					1	0	0	1	1	1	1
2347	cotton					151.2	1	0	0	1	0	1	1
2347	cotton						1	0	0	1	0	1	1
2347	cotton						1	0	0	1	0	1	1
2347	cotton						1	0	0	1	0	1	1
2347	cotton				-30.8		1	0	0	1	0	1	1
2347	cotton						1	0	0	1	0	1	1
2347	cotton						1	0	0	1	0	1	1
2347	cotton	30.4					1	0	0	1	0	1	1
2347	cotton				-8.9		1	0	0	1	0	1	1
2347	cotton						1	0	0	1	0	1	1
2361	cotton		-68.1				1	0	0	1	0	1	1
2361	cotton		-31.4				1	1	0	1	0	1	1
2361	cotton			-48.0			1	0	0	1	0	1	1
2361	cotton		-31.5				1	0	0	1	0	1	1
2361	cotton						1	0	0	1	0	1	1
2361	cotton		-22.8				1	0	0	1	0	1	1
2361	cotton						1	1	0	1	0	1	1
2361	cotton		-32.7				1	0	0	1	0	1	1
2361	cotton		-54.7				1	0	0	1	0	1	1
2361	cotton						1	0	0	1	0	1	1
2361	cotton						1	0	0	1	0	1	1
2361	cotton						1	1	0	1	0	1	1
2361	cotton						1	0	0	1	0	1	1
2361	cotton						1	1	0	1	0	1	1
2362	maize							1	1	0	0	0	0
2362	cotton							1	1	0	0	0	0
2362	soybean							1	1	0	0	0	0
2372	cotton	60.5					1	0	0	1	0	1	1
2372	cotton	18.3					1	0	0	1	0	1	1
2372	cotton	55.0					1	0	0	1	0	1	1
2372	cotton		-46.6				1	0	0	1	0	1	1
2372	cotton	33.3					1	0	0	1	0	1	1
2372	cotton		-54.8				1	0	0	1	0	1	1
2372	cotton		-48.6				1	0	0	1	0	1	1

2372	cotton	-9.7					1	0	0	1	0	1	1
2372	cotton		-42.0				1	0	0	1	0	1	1
2372	cotton		-57.1				1	0	0	1	0	1	1
2383	maize						1	1	0	1	1	1	1
2383	maize	27.5					1	0	0	1	1	1	1
2383	maize	61.1					1	0	0	1	1	1	1
2383	maize						1	0	0	1	1	1	1
2383	maize	29.9					1	0	0	1	1	1	1
2383	maize						1	0	0	1	1	1	1
2383	maize	29.7					1	0	0	1	1	1	1
2383	maize	55.1					1	0	0	1	1	1	1
2383	maize	40.3					1	0	0	1	1	1	1
2383	maize					13.6	1	0	0	1	1	1	1
2384	cotton						1	0	0	1	0	1	1
2384	cotton						1	0	0	1	0	1	1
2384	cotton						1	0	0	1	0	1	1
2384	cotton						1	0	0	1	0	1	1
2389	maize	16.4					0	0	0	0	0	1	1
2389	maize	20.7					1	0	0	0	0	1	1
2399	maize	33.1					1	1	0	0	0	0	1
2399	maize	12.4					1	0	0	0	0	0	1
2399	maize		-44.4				1	0	0	0	0	0	1
2409	cotton	5.1					1	0	0	1	1	1	1
2409	cotton	3.8					1	0	0	1	1	1	1
2410	cotton						1	1	0	1	0	1	1
2410	cotton						1	1	0	1	0	1	1
2410	cotton						1	1	0	1	0	1	1
2410	cotton						1	1	0	1	0	1	1
2414	soybean	12.5					0	1	1	0	0	0	0
2414	soybean	30.2					0	1	1	0	0	0	0
2414	soybean	7.0					0	1	1	0	0	0	0
2433	maize	25.5					1	1	0	1	0	1	1
2433	maize						1	1	0	1	0	1	1
2433	maize		-74.5				1	1	0	1	0	1	1
2433	maize						1	1	0	1	0	1	1
2433	maize						1	1	0	1	0	1	1
2433	maize						1	1	0	1	0	1	1
2433	maize		33.0				1	1	0	1	0	1	1
2433	maize	14.7					1	1	0	1	0	1	1
2433	maize					53.8	1	1	0	1	0	1	1
2433	maize		-65.3				1	1	0	1	0	1	1
2433	maize		73.8				1	1	0	1	0	1	1
2433	maize					78.3	1	1	0	1	0	1	1
2435	cotton						1	0	0	1	0	0	0
2435	cotton	28.1					1	0	0	1	0	0	0
2435	cotton						1	0	0	1	0	0	0
2435	cotton		-21.2				1	0	0	1	0	0	0
2435	cotton						1	0	0	1	0	0	0
2435	cotton					35.0	1	0	0	1	0	0	0
2435	cotton			-15.6			1	0	0	1	0	0	0
2435	cotton				10.7		1	0	0	1	0	0	0
2435	cotton						1	0	0	1	0	0	0
2443	cotton						1	0	0	1	0	1	1
2443	cotton	4.2					1	0	0	1	0	1	1
2443	cotton						1	0	0	1	0	1	1
2443	cotton						1	0	0	1	0	1	1
2443	cotton						1	0	0	1	0	1	1
2443	cotton						1	0	0	1	0	1	1
2454	cotton						1	0	0	1	0	0	0
2454	cotton						1	0	0	1	0	0	0
2454	cotton		-58.2				1	0	0	1	0	0	0
2454	cotton						1	0	0	1	0	0	0
2476	cotton	12.4					1	1	0	1	0	0	0
2476	cotton						1	1	0	1	0	0	0
2476	cotton	14.2					1	1	0	1	0	0	0
2476	cotton						1	1	0	1	0	0	0
2476	cotton						1	1	0	1	0	0	0

2476	cotton	19.0					1	1	0	1	0	0	0
2487	cotton	19.3					1	0	0	1	1	1	1
2487	cotton	48.5					1	0	0	1	1	1	1
2488	maize						1	0	0	1	0	1	1
2488	maize		-28.7				1	0	0	1	0	1	1
2488	maize						1	0	0	1	0	1	1
2488	maize						1	0	0	1	0	1	1
2488	maize	34.3					1	0	0	1	0	1	1
2488	maize						1	0	0	1	0	1	1
2488	maize					88.8	1	0	0	1	0	1	1
2488	maize						1	0	0	1	0	1	1
2488	maize						1	0	0	1	0	1	1
2488	maize						1	0	0	1	0	1	1
2488	maize						1	0	0	1	0	1	1
2502	cotton					4.6	1	0	0	1	0	1	1
2502	cotton	6.8					1	0	0	1	0	1	1
2502	cotton	-1.7					1	0	0	1	0	1	1
2502	cotton				-3.1		1	0	0	1	0	1	1
2502	cotton	5.0					1	0	0	1	0	1	1
2502	cotton					11.0	1	0	0	1	0	1	1
2502	cotton	4.8					1	0	0	1	0	1	1
2521	cotton						1	0	0	1	1	0	0
2523	maize	0.1					1	0	0	0	0	1	1
2523	maize	0.3					0	0	0	0	0	1	1
2529	cotton		4.2				1	0	0	1	0	1	1
2529	cotton						1	0	0	1	0	1	1
2529	cotton						1	1	0	1	0	1	1
2529	cotton					102.0	1	0	0	1	0	1	1
2529	cotton		-37.0				1	0	0	1	0	1	1
2529	cotton	35.3					1	0	0	1	0	1	1
2529	cotton					93.2	1	0	0	1	0	1	1
2529	cotton	36.7					1	0	0	1	0	1	1
2529	cotton						1	0	0	1	0	1	1
2529	cotton						1	1	0	1	0	1	1
2551	cotton						1	0	0	1	0	1	1
2551	cotton						1	0	0	1	0	1	1
2551	cotton						1	0	0	1	0	1	1
2551	cotton						1	0	0	1	0	1	1
2551	cotton				8.2		1	0	0	1	0	1	1
2551	cotton						1	0	0	1	0	1	1
2551	cotton						1	0	0	1	0	1	1
2551	cotton						1	0	0	1	0	1	1
2551	cotton						1	0	0	1	0	1	1
2551	cotton			-33.3			1	0	0	1	0	1	1
2555	maize	34.7					1	0	0	1	0	0	1
2555	maize		-80.0				1	0	0	1	0	0	1
2555	maize						1	0	0	1	0	0	1
2555	maize						1	0	0	1	0	0	1
2555	maize		-66.2				1	0	0	1	0	0	1
2555	maize	25.5					1	0	0	1	0	0	1
2555	maize						1	0	0	1	0	0	1
2555	maize						1	0	0	1	0	0	1
2555	maize						1	1	0	1	0	0	1
2555	maize						1	0	0	1	0	0	1
2555	maize						1	1	0	1	0	0	1
2555	maize	12.6					1	0	0	1	0	0	1
2555	maize	20.4					1	0	0	1	0	0	1
2555	maize						1	0	0	1	0	0	1
2555	maize						1	0	0	1	0	0	1
2555	maize		-74.7				1	0	0	1	0	0	1
2555	maize						1	0	0	1	0	0	1
2555	maize						1	0	0	1	0	0	1
2555	maize						1	1	0	1	0	0	1
2555	maize						1	0	0	1	0	0	1
2555	maize						1	0	0	1	0	0	1
2555	maize						1	1	0	1	0	0	1
2555	maize						1	1	0	1	0	0	1

2555	maize						1	0	0	1	0	0	1
2555	maize		-74.2				1	0	0	1	0	0	1
2555	maize						1	1	0	1	0	0	1
2556	maize		0.0				1	0	0	0	0	0	1
2556	maize	20.0					1	0	0	0	0	0	1
2556	maize						1	1	0	0	0	0	1
2556	maize					23.0	1	1	0	0	0	0	1
2556	maize	23.0					1	1	0	0	0	0	1
2562	cotton	44.7					1	0	0	1	0	0	1
2562	cotton	61.0					1	0	0	1	0	0	1
2562	cotton			-21.1			1	0	0	1	0	0	1
2562	cotton					40.9	1	0	0	1	0	0	1
2562	cotton					75.7	1	0	0	1	0	0	1
2562	cotton			-27.8			1	0	0	1	0	0	1
2562	cotton						1	0	0	1	0	0	1
2562	cotton						1	0	0	1	0	0	1
2571	cotton			-26.8			1	0	0	1	0	0	1
2571	cotton			-47.1			1	0	0	1	0	0	1
2571	cotton	5.9					1	0	0	1	0	0	1
2571	cotton						1	0	0	1	0	0	1
2571	cotton						1	0	0	1	0	0	1
2571	cotton				-4.2		1	0	0	1	0	0	1
2571	cotton					17.9	1	0	0	1	0	0	1
2571	cotton	39.4					1	0	0	1	0	0	1
2571	cotton					77.7	1	0	0	1	0	0	1
2571	cotton						1	0	0	1	0	0	1
2571	cotton						1	0	0	1	0	0	1
2571	cotton						1	0	0	1	0	0	1
2571	cotton						1	0	0	1	0	0	1
2571	cotton				4.5		1	0	0	1	0	0	1
2571	cotton			-21.1			1	0	0	1	0	0	1
2571	cotton						1	0	0	1	0	0	1
2571	cotton						1	0	0	1	0	0	1
2571	cotton			-52.8			1	0	0	1	0	0	1
2577	cotton						1	0	0	1	0	1	1
2577	cotton						1	0	0	1	0	1	1
2577	cotton				-9.6		1	0	0	1	0	1	1
2577	cotton	39.2					1	1	0	1	0	1	1
2577	cotton						1	0	0	1	0	1	1
2577	cotton			-18.2			1	0	0	1	0	1	1
2577	cotton				1.5		1	0	0	1	0	1	1
2577	cotton	-29.8					1	0	0	1	0	1	1
2577	cotton						1	0	0	1	0	1	1
2577	cotton						1	0	0	1	0	1	1
2577	cotton						1	0	0	1	0	1	1
2577	cotton						1	0	0	1	0	1	1
2577	cotton	41.2					1	1	0	1	0	1	1
2577	cotton						1	0	0	1	0	1	1
2577	cotton						1	0	0	1	0	1	1
2577	cotton						1	0	0	1	0	1	1
2577	cotton						1	0	0	1	0	1	1
2577	cotton				17.0		1	0	0	1	0	1	1
2577	cotton	31.6					1	0	0	1	0	1	1
2577	cotton						1	0	0	1	0	1	1
2577	cotton				11.5		1	0	0	1	0	1	1
2580	cotton						1	0	0	1	0	1	1
2580	cotton						1	0	0	1	0	1	1
2580	cotton							1	0	1	0	1	1
2580	cotton	64.9					1	0	0	1	0	1	1
2580	cotton	11.2					1	1	0	1	0	1	1
2580	cotton						1	0	0	1	0	1	1
2580	cotton				-8.3		1	0	0	1	0	1	1
2580	cotton						1	0	0	1	0	1	1
2580	cotton						1	0	0	1	0	1	1
2580	cotton	36.2					1	1	0	1	0	1	1
2580	cotton						1	0	0	1	0	1	1
2601	cotton				11.9		1	0	0	1	0	1	1

2601	cotton						1	0	0	1	0	1	1
2601	cotton						1	0	0	1	0	1	1
2601	cotton						1	0	0	1	0	1	1
2601	cotton						1	0	0	1	0	1	1
2601	cotton						1	0	0	1	0	1	1
2601	cotton	43.4					1	0	0	1	0	1	1
2601	cotton			-23.9			1	0	0	1	0	1	1
2632	maize					16.5	1	1	0	0	0	1	1
2632	maize	17.1					1	1	0	0	0	1	1
2702	soybean						0	0	0	0	0	0	0
2702	soybean	1.2					0	0	0	0	0	0	0
2702	soybean						0	0	0	0	0	0	0
2702	soybean	3.1					0	0	0	0	0	0	0
2771	maize					30.0	1	0	0	1	0	1	1
2771	maize					27.6	0	0	0	1	0	1	1
2771	maize			-68.8			0	0	0	1	0	1	1
2771	maize				-5.1		0	0	0	1	0	1	1
2771	maize	4.9					1	0	0	1	0	1	1
2771	maize				0.4		1	0	0	1	0	1	1
2771	maize			-90.8			1	0	0	1	0	1	1
2771	maize							1	0	1	0	1	1
2771	maize	0.0					0	0	0	1	0	1	1
2776	cotton			-29.9			1	0	0	0	0	0	0
2776	cotton		-53.5				1	0	0	0	0	0	0
2776	cotton	3.4					1	0	0	0	0	0	0
2778	cotton		-64.0				1	0	0	0	0	0	0
2778	cotton			-35.1			1	0	0	0	0	0	0
2778	cotton	5.0					1	0	0	0	0	0	0
2778	cotton			-38.2			1	0	0	0	0	0	0
2779	maize						1	0	0	0	0	0	1
2779	maize	2.0					1	0	0	0	0	0	1
2779	soybean						0	0	0	0	0	0	1
2779	soybean	-3.6					0	0	0	0	0	0	1
2779	soybean			-23.6			0	0	0	0	0	0	1
2779	maize						1	0	0	0	0	0	1
2779	soybean			-22.0			0	0	0	0	0	0	1
2780	soybean			-26.1			0	0	0	0	0	0	0
2780	soybean						0	0	0	0	0	0	0
2780	maize						1	0	0	0	0	0	0
2780	maize				12.5		1	0	0	0	0	0	0
2780	soybean				-6.4		0	0	0	0	0	0	0
2780	soybean			-26.4			0	0	0	0	0	0	0
2780	maize						1	0	0	0	0	0	0
2801	soybean	13.0					0	0	0	0	0	1	1
2801	soybean	-14.7					0	0	0	0	0	1	1
2801	soybean				2.5		0	0	0	0	0	1	1
2801	soybean				3.7		0	0	0	0	0	1	1
2801	soybean				6.2		0	0	0	0	0	1	1
2801	soybean	18.2					0	0	0	0	0	1	1
2801	soybean	15.4					0	0	0	0	0	1	1
2801	soybean				4.9		0	0	0	0	0	1	1
2827	cotton		-24.0				1	0	0	1	0	1	1
2827	cotton				22.3		1	0	0	1	0	1	1
2827	cotton			-27.2			1	0	0	1	0	1	1
2827	cotton						1	0	0	1	0	1	1
2827	cotton	23.4					1	0	0	1	0	1	1
2827	cotton						1	0	0	1	0	1	1
2836	cotton			-18.0			1	1	0	1	0	0	0
2836	cotton				5.9		1	1	0	1	0	0	0
2836	cotton				36.5		1	0	0	1	0	0	0
2836	cotton	28.5					1	0	0	1	0	0	0
2836	cotton				3.3		1	1	0	1	0	0	0
2836	cotton						1	0	0	1	0	0	0
2836	cotton	46.3					1	0	0	1	0	0	0
2836	cotton	44.6					1	0	0	1	0	0	0
2836	cotton						1	1	0	1	0	0	0
2836	cotton						1	0	0	1	0	0	0

2836	cotton						1	0	0	1	0	0	0
2836	cotton						1	1	0	1	0	0	0
2836	cotton						1	0	0	1	0	0	0
2836	cotton			-28.2			1	0	0	1	0	0	0
2836	cotton				13.5		1	1	0	1	0	0	0
2836	cotton					58.1	1	0	0	1	0	0	0
2836	cotton				5.6		1	0	0	1	0	0	0
2836	cotton						1	1	0	1	0	0	0
2836	cotton						1	0	0	1	0	0	0
2836	cotton					106.9	1	1	0	1	0	0	0
2836	cotton						1	0	0	1	0	0	0
2836	cotton						1	0	0	1	0	0	0
2836	cotton					164.1	1	0	0	1	0	0	0
2836	cotton						1	1	0	1	0	0	0
2836	cotton					75.8	1	0	0	1	0	0	0
2836	cotton					247.1	1	0	0	1	0	0	0
2836	cotton						1	1	0	1	0	0	0
2836	cotton				13.7		1	0	0	1	0	0	0
2836	cotton						1	0	0	1	0	0	0
2836	cotton						1	0	0	1	0	0	0
2836	cotton						1	0	0	1	0	0	0
2836	cotton			-21.3			1	0	0	1	0	0	0
2836	cotton			-28.5			1	1	0	1	0	0	0
2836	cotton						1	0	0	1	0	0	0
2836	cotton						1	1	0	1	0	0	0
2836	cotton				13.7		1	0	0	1	0	0	0
2836	cotton	43.7					1	1	0	1	0	0	0
2836	cotton						1	0	0	1	0	0	0
2836	cotton						1	1	0	1	0	0	0
2836	cotton					74.5	1	1	0	1	0	0	0
2836	cotton			-54.5			1	0	0	1	0	0	0
2836	cotton	35.4					1	1	0	1	0	0	0
2836	cotton						1	0	0	1	0	0	0
2836	cotton					143.2	1	1	0	1	0	0	0
2836	cotton	24.7					1	1	0	1	0	0	0
2836	cotton						1	1	0	1	0	0	0
2836	cotton						1	1	0	1	0	0	0
2836	cotton						1	0	0	1	0	0	0
2836	cotton	35.4					1	0	0	1	0	0	0
2836	cotton						1	1	0	1	0	0	0
2836	cotton						1	0	0	1	0	0	0
2836	cotton			-44.8			1	1	0	1	0	0	0
2836	cotton						1	1	0	1	0	0	0
2836	cotton						1	1	0	1	0	0	0
2836	cotton						1	0	0	1	0	0	0
2836	cotton			-18.0			1	0	0	1	0	0	0
2875	cotton						1	0	0	1	0	0	0
2875	cotton						1	1	0	1	0	0	0
2875	cotton	-17.4					1	0	0	1	0	0	0
2875	cotton						1	1	0	1	0	0	0
2875	cotton						1	1	0	1	0	0	0
2875	cotton						1	1	0	1	0	0	0
2875	cotton						1	1	0	1	0	0	0
2875	cotton			-5.0			1	0	0	1	0	0	0
2875	cotton						1	0	0	1	0	0	0
2875	cotton						1	0	0	1	0	0	0
2875	cotton						1	1	0	1	0	0	0
2875	cotton						1	1	0	1	0	0	0
2875	cotton						1	0	0	1	0	0	0
2875	cotton						1	0	0	1	0	0	0
2875	cotton		-50.0				1	0	0	1	0	0	0
2875	cotton						1	1	0	1	0	0	0
2875	cotton						1	1	0	1	0	0	0
2875	cotton	-25.0					1	0	0	1	0	0	0
2875	cotton						1	1	0	1	0	0	0
2875	cotton						1	1	0	1	0	0	0
2875	cotton						1	1	0	1	0	0	0

2875	cotton					1	1	0	1	0	0	0
2875	cotton			-58.4		1	0	0	1	0	0	0
2875	cotton					1	0	0	1	0	0	0
2896	maize	7.1				1	0	0	0	0	0	0
2896	soybean	-3.2				0	0	0	0	0	0	0
2896	maize	19.6				1	0	0	0	0	0	0
2896	soybean	-0.8				0	0	0	0	0	0	0
2896	maize	-0.1				1	0	0	0	0	0	0
2896	soybean	-4.2				0	0	0	0	0	0	0
2896	soybean	-6.1				0	0	0	0	0	0	0
2896	maize	14.8				1	0	0	0	0	0	0
2896	soybean	2.8				0	0	0	0	0	0	0
2896	maize	14.2				1	0	0	0	0	0	0
2896	soybean	-1.2				0	0	0	0	0	0	0
2896	soybean	17.6				0	0	0	0	0	0	0
2896	maize	5.8				1	0	0	0	0	0	0
2896	maize	13.0				1	0	0	0	0	0	0
2896	soybean	-10.8				0	0	0	0	0	0	0
2896	maize	-4.2				1	0	0	0	0	0	0
2896	maize	23.6				1	0	0	0	0	0	0
2896	maize	5.2				1	0	0	0	0	0	0
2896	soybean	15.1				0	0	0	0	0	0	0
2896	soybean	3.9				0	0	0	0	0	0	0
2896	soybean	28.3				0	0	0	0	0	0	0
2896	soybean	8.7				0	0	0	0	0	0	0
2896	soybean	2.6				0	0	0	0	0	0	0
2896	maize	14.1				1	0	0	0	0	0	0
2896	soybean	-3.9				0	0	0	0	0	0	0
2896	maize	-17.4				1	0	0	0	0	0	0
2896	maize	-40.7				1	0	0	0	0	0	0
2896	soybean	15.0				0	0	0	0	0	0	0
2896	soybean	0.0				0	0	0	0	0	0	0
2896	maize	12.1				1	0	0	0	0	0	0
2896	maize	9.7				1	0	0	0	0	0	0
2930	cotton		-71.3			1	0	0	1	0	0	1
2930	cotton	46.0				1	0	0	1	0	0	1
2930	cotton	26.6				1	0	0	1	0	0	1
2930	cotton		-78.5			1	0	0	1	0	0	1
2930	cotton	58.2				1	0	0	1	0	0	1
2930	cotton				211.7	1	0	0	1	0	0	1
2930	cotton			-80.5		1	0	0	1	0	0	1
2930	cotton			-71.5		1	0	0	1	0	0	1
2930	cotton		-71.6			1	0	0	1	0	0	1
2930	cotton				46.0	1	0	0	1	0	0	1
2930	cotton		-63.7			1	0	0	1	0	0	1
2930	cotton				162.6	1	0	0	1	0	0	1
2930	cotton	49.9				1	0	0	1	0	0	1
2930	cotton			-81.7		1	0	0	1	0	0	1
2930	cotton		-72.3			1	0	0	1	0	0	1
2930	cotton			-88.1		1	0	0	1	0	0	1
2930	cotton		-68.2			1	0	0	1	0	0	1
2930	cotton		-59.5			1	0	0	1	0	0	1
2930	cotton			-79.4		1	0	0	1	0	0	1
2930	cotton	32.8				1	0	0	1	0	0	1
2930	cotton			-62.0		1	0	0	1	0	0	1
2930	cotton			-78.5		1	0	0	1	0	0	1
2930	cotton				89.8	1	0	0	1	0	0	1
2930	cotton				8.3	1	0	0	1	0	0	1
2930	cotton	41.8				1	0	0	1	0	0	1
2984	cotton		63.2			1	0	0	1	0	0	0
2984	cotton		-57.9			1	0	0	1	0	0	0
2984	cotton	23.7				1	0	0	1	0	0	0
2984	cotton		-3.6			1	0	0	1	0	0	0
2984	cotton	25.0				1	0	0	1	0	0	0
2984	cotton	82.3				1	0	0	1	0	0	0
2984	cotton		-13.6			1	0	0	1	0	0	0
3022	maize	29.7				1	0	0	0	0	0	0

3022	maize	7.5					1	0	0	0	0	0	0
3022	maize	9.4					1	0	0	0	0	0	0
3022	maize				-2.8		1	0	0	0	0	0	0
3022	maize						1	0	0	0	0	0	0
3022	maize						1	0	0	0	0	0	0
3022	maize	8.2					1	0	0	0	0	0	0
3047	cotton						1	0	0	1	0	1	1
3047	cotton					69.0	1	0	0	1	0	1	1
3047	cotton	34.2					1	0	0	1	0	1	1
3047	cotton	60.2					1	1	0	1	0	1	1
3047	cotton		-38.4				1	0	0	1	0	1	1
3047	cotton			-40.9			1	0	0	1	0	1	1
3047	cotton	44.2					1	1	0	1	0	1	1
3047	cotton						1	0	0	1	0	1	1
3047	cotton						1	0	0	1	0	1	1
3047	cotton						1	0	0	1	0	1	1
3047	cotton		-50.4				1	0	0	1	0	1	1
3047	cotton				16.8		1	0	0	1	0	1	1
3096	soybean						0	0	0	0	0	0	0
3096	soybean		-1.6				0	0	0	0	0	0	0
3096	cotton			-6.8			1	0	0	0	0	0	0
3096	cotton			-9.1			1	0	0	0	0	0	0
3096	soybean			-8.0			0	0	0	0	0	0	0
3128	cotton						1	0	0	1	0	0	0
3128	cotton				17.3		1	0	0	1	0	0	0
3128	cotton						1	0	0	1	0	0	0
3128	cotton						1	0	0	1	0	0	0
3128	cotton						1	0	0	1	0	0	0
3128	cotton				10.4		1	0	0	1	0	0	0
3128	cotton						1	0	0	1	0	0	0
3128	cotton	5.4					1	0	0	1	0	0	0
3128	cotton						1	0	0	1	0	0	0
3128	cotton				8.1		1	0	0	1	0	0	0
3128	cotton	-34.8					1	0	0	1	0	0	0
3128	cotton	3.4					1	0	0	1	0	0	0
3128	cotton					-8.9	1	0	0	1	0	0	0
3133	cotton						1	0	0	1	0	1	1
3133	cotton						1	0	0	1	0	1	1
3133	cotton	20.7					1	0	0	1	0	1	1
3133	cotton	29.6					1	0	0	1	0	1	1
3133	cotton						1	0	0	1	0	1	1
3133	cotton						1	0	0	1	0	1	1
3133	cotton				21.2		1	0	0	1	0	1	1
3133	cotton					27.2	1	0	0	1	0	1	1
3133	cotton				20.4		1	0	0	1	0	1	1
3133	cotton					41.0	1	0	0	1	0	1	1
3152	cotton	0.0					1	0	0	1	0	0	0
3152	cotton			-7.9			1	0	0	1	0	0	0
3152	cotton				15.4		1	0	0	1	0	0	0
3152	cotton					-5.1	1	0	0	1	0	0	0
3153	cotton						1	0	0	1	0	0	0
3153	cotton	-15.4					1	0	0	1	0	0	0
3153	cotton						1	0	0	1	0	0	0
3153	cotton					-48.3	1	0	0	1	0	0	0
3153	cotton						1	0	0	1	0	0	0
3153	cotton	-13.6					1	0	0	1	0	0	0
3153	cotton			-14.2			1	0	0	1	0	0	0
3153	cotton				20.8		1	0	0	1	0	0	0
3153	cotton	-16.7					1	0	0	1	0	0	0
3153	cotton						1	0	0	1	0	0	0
3153	cotton						1	0	0	1	0	0	0
3153	cotton					-38.6	1	0	0	1	0	0	0
3227	cotton	6.1					1	0	0	1	0	1	1
3227	cotton						1	0	0	1	0	1	1
3227	cotton						1	0	0	1	0	1	1
3239	cotton						0	1	0	0	0	0	0
3239	cotton					-5.4	0	0	0	0	0	0	0

3239	cotton	-3.4					0	0	0	0	0	0	0
3239	cotton			-3.5			0	0	0	0	0	0	0
3239	cotton		-21.3				0	0	0	0	0	0	0
3239	cotton				0.0		0	0	0	0	0	0	0
3239	cotton						0	0	0	0	0	0	0
3239	cotton			-10.9			0	0	0	0	0	0	0
3243	cotton						1	0	0	1	0	1	1
3243	cotton						1	0	0	1	0	1	1
3243	cotton						1	0	0	1	0	1	1
3243	cotton						1	0	0	1	0	1	1
3243	cotton	-7.7					1	0	0	1	0	1	1
3243	cotton						1	0	0	1	0	1	1
3243	cotton	-4.4					1	0	0	1	0	1	1
3243	cotton						1	0	0	1	0	1	1
3243	cotton						1	0	0	1	0	1	1
3243	cotton			-8.2			1	0	0	1	0	1	1
3243	cotton						1	0	0	1	0	1	1
3243	cotton						1	0	0	1	0	1	1
3243	cotton	-10.0					1	0	0	1	0	1	1
3243	cotton	-5.6					1	0	0	1	0	1	1
3243	cotton						1	0	0	1	0	1	1
3243	cotton						1	0	0	1	0	1	1
3243	cotton			-63.5			1	0	0	1	0	1	1
3243	cotton			-22.7			1	0	0	1	0	1	1
3243	cotton			-39.5			1	0	0	1	0	1	1
3243	cotton						1	0	0	1	0	1	1
3246	maize	34.3					1	0	0	1	0	1	1
3246	maize			-51.9			1	0	0	1	0	1	1
3246	maize						1	0	0	1	0	1	1
3246	maize						1	0	0	1	0	1	1
3246	maize				-4.4		1	0	0	1	0	1	1
3246	maize	25.2					1	1	0	1	0	1	1
3247	cotton	17.6					1	0	0	1	0	0	0
3247	cotton						1	0	0	1	0	0	0
3247	cotton	9.2					1	0	0	1	0	0	0
3247	cotton						1	0	0	1	0	0	0
3247	cotton				-0.1		1	0	0	1	0	0	0
3247	cotton				-8.5		1	0	0	1	0	0	0
3247	cotton					131.0	1	0	0	1	0	0	0
3247	cotton				-22.2		1	0	0	1	0	0	0
3247	cotton					-4.8	1	0	0	1	0	0	0
3247	cotton	74.6					1	0	0	1	0	0	0
3247	cotton						1	0	0	1	0	0	0
